# Values of integration between lipidomics and clinical phenomes in patients with acute lung infection, pulmonary embolism, or acute exacerbation of chronic pulmonary diseases: a preliminary study

**DOI:** 10.1186/s12967-019-1898-z

**Published:** 2019-05-20

**Authors:** Danyan Gao, Linlin Zhang, Dongli Song, Jiapei Lv, Linyan Wang, Shuang Zhou, Yanjun Li, Tao Zeng, Yiming Zeng, Jiaqiang Zhang, Xiangdong Wang

**Affiliations:** 1 Department of Pulmonary Diseases, The First Hospital of Wenzhou Medical University, Wenzhou, Zhejiang China; 2 Zhongshan Hospital Institute for Clinical Science, Shanghai Institute of Clinical Bioinformatics, Shanghai Engineering Research for AI Technology for Cardiopulmonary Diseases, Center for Tumor Diagnosis and Therapy, Jinshan Hospital, Shanghai Medical College, Fudan University, Shanghai, China; 3 Clinical Center for Molecular Diagnosis and Therapy, The Second Hospital of Fujian Medical University, Quanzhou, Fujian China; 4 Department of Anesthesiology, Center for Clinical Single Cell Biomedicine, Henan Provincial People’s Hospital, People’s Hospital of Zhengzhou University, Zhengzhou, China; 5 Key Laboratory of Systems Biology, Institute of Biochemistry and Cell Biology, Chinese Academy Science, Shanghai, China

**Keywords:** Acute pneumonia, Pulmonary embolism, Chronic pulmonary disease, Phenomes, Lipidomics

## Abstract

**Background:**

The morbidity and mortality of patients with critical illnesses remain high in pulmonary critical care units and a poorly understood correlation between alterations of lipid elements and clinical phenomes remain unelucidated.

**Methods:**

In the present study, we investigated plasma lipidomic profiles of 30 patients with severe acute pneumonia (SAP), acute pulmonary embolism (APE), and acute exacerbation of chronic pulmonary diseases (AECOPD) or 15 healthy with the aim to compare disease specificity of lipidomic patterns. We defined the specificity of lipidomic profiles in SAP by comparing it to both APE and AECOPD. Analysis of the correlation between altered lipid elements and clinical phenotypes using the lipid-QTL model was then carried out.

**Results:**

We integrated lipidomic profiles with clinical phenomes measured by score values from the digital evaluation score system and found phenome-associated lipid elements to identify disease-specific lipidomic profiling. The present study demonstrates that lipidomic profiles of patients with acute lung diseases are different from healthy lungs, and there are also disease-specific portions of lipidomics among SAP, APE, or AECOPD. The comprehensive profiles of clinical phenomes or lipidomics are valuable in describing the disease specificity of patient phenomes and lipid elements. The combination of clinical phenomes with lipidomic profiles provides more detailed disease-specific information on panels of lipid elements When compared to the use of each separately.

**Conclusions:**

Integrating biological functions with disease specificity, we believe that clinical lipidomics may create a new alternative way to understand lipid-associated mechanisms of critical illnesses and develop a new category of disease-specific biomarkers and therapeutic targets.

**Electronic supplementary material:**

The online version of this article (10.1186/s12967-019-1898-z) contains supplementary material, which is available to authorized users.

## Background

Acute lung injury is a major challenge and cause of patient morbidity and mortality in pulmonary critical care units, as an early stage of acute respiratory distress syndrome (ARDS), although molecular diagnosis and therapy are still lacking due to the complex pathogenesis, severity, and systemic responses [1]. Acute infection is one of common factors that can induce the exacerbation of chronic lung diseases. Chen et al., initially identified disease-specific dynamic biomarkers for severe pneumonia or severe pneumonia-associated-ARDS by integrating proteomic profiles of inflammatory mediators with clinical informatics as part of clinical bioinformatics [2]. They found that specific protein-based biomarkers comparing diseased tissue with healthy tissue and diseased tissue and diseased tissue had a significant correlation with clinical phenomes measured by Digital Evaluation Score System (DESS) scores. Shi et al. identified specific immunomodulatory mediators by evaluating dynamic genomic and proteomic profiles of peripheral blood mononuclear cells and plasma in patients with acute exacerbation of chronic obstructive pulmonary disease (AECOPD) and found a complex network of AECOPD- or COPD-specific immunomodulatory mediators [3]. In addition to changes of genomic and proteomic profiles, alterations of systemic metabolisms are also another important factor which can influence disease severity, duration, progression, and patient response to therapy, although the metabolism has been ignored in understanding of molecular mechanisms in the development of acute and chronic pulmonary diseases.

Clinical lipidomics is a new integrative approach to identify the disease-specific correlation and regulation between a large scale of lipid elements measured in liquid biopsies from patients with their clinical phenomes [4]. Clinical lipidomics has been suggested as a novel approach in discovering new categories of disease-specific biomarkers or therapeutic targets and could play a key role in improving our understanding of molecular mechanisms in disease metabolisms [5]. However, challenges still remain to be faced and overcome in prior to clinical practice [6]. Lv et al. investigated regulatory mechanisms of lipidomic profiles in lung cancer subtypes by measuring plasma lipidomes between healthy patients and patients with lung cancer, squamous cell carcinomas, adenocarcinomas, and small cell lung cancers, and initially correct lipidomic and genomic profiles of lipid-associated enzymes and proteins by integrating the data of large-scale genome screening [7]. The integration of lipidomics with genomics can be a new approach in identifying disease-specific diagnostic biomarkers and therapeutic targets.

In the present study, we investigated plasma lipidomic profiles of patients with severe acute pneumonia (SAP), acute pulmonary embolism (APE), and AECOPD with the aim to compare disease specificity of lipidomic patterns. We defined the specificity of lipidomic profiles in SAP by comparing it to both APE and AECPOD. Analysis of the correlation between altered lipid elements and clinical phenotypes using the lipid-QTL model was then carried out.

## Methods and materials

### Patient population

**Fig. 1:**
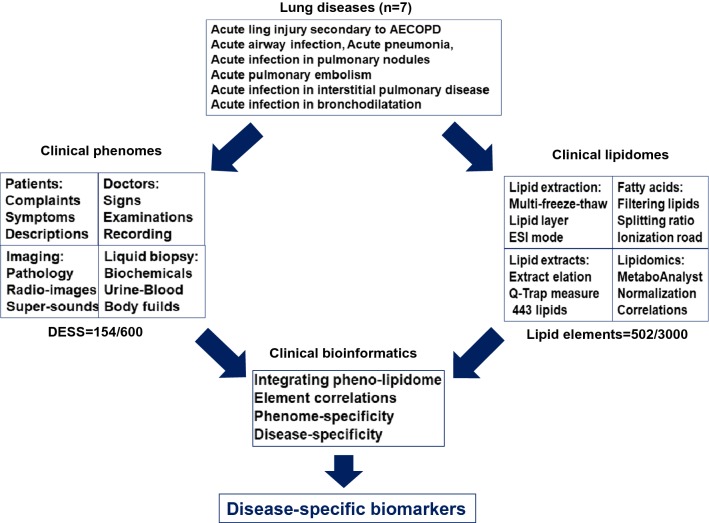
Work-flow of integration processes between clinical phenomes and lipidomic profiles using clinical bioinformatics for the identification of disease-specific biomarkers

Patients that were selected in the study had either SAP, APE, or AECOPD. The present study was approved by the Ethical Evaluation Committee of Zhongshan Hospital and designed using a case–control approach. The patients gave informed consent for lipids analysis and the ethical code is B2018-187. The study involved 30 patients (11 SAP, 7 APE and 12 AECOPD) and 15 healthy people. SAP was defined according to the previous description [8, 9] and APE was diagnosed as described previously [10]. Patient medical history included admittance to the hospital due to an acute infection associated with AECOPD [11]. Patients were recruited into the study between October 2016 and March 2017. Healthy controls enrolled were blood donors at Zhongshan Hospital. Subjects accompanied with more than one respiratory disease, or any family history of lung disease, were excluded. Plasma was harvested from healthy controls (10 female/5 male) and patients with SAP, ARE, or AECOPD on day one when they entered the hospital. Informed consent was given by the subjects themselves before they underwent lung function tests, high-resolution computed tomography and blood collection. The details of the study design are explained in Fig. 1.

### Digital evaluation score system

The Digital Evaluation Score System (DESS) is a score index used to translate clinical descriptive information of each phenome into clinical informatics, as described previously [12]. Using this instrument, we considered clinical phenomes (e.g. symptoms and signs, biochemical analyses and clinical imaging) for patients with SAP, APE, or AECOPD, respectively. When assessing severity each component was assigned a score of 0, 1, 2 or 4 with the score of 4 indicating far above normal range or a much severe condition, and 0 as the minimum value that indicates that severity is within normal physiological range. After compiling patient data, we added the points for each variable. The total DESS scores ranged from 0 to 584 points, with a higher score indicating a more severe condition. Patients were scored on the day when their blood samples were collected. Of the 500 clinical phenomes scored and collected in each of three lung diseases, 146 clinical phenomes were listed in Additional file 1: Table S1 and the terminal data was derived from the sum of each phenome score.

### Lipid extraction for mass spectrometry analysis

A total of 200 μl plasma was taken into a glass tube and managed, as reported previously [13]. Then, 10 μl internal standard was added into each glass tube and then poured into 5 ml of methanol:chloroform:formic acid (10:10:1). This mixture was shaken vigorously and incubated at − 20 °C overnight. 2 ml of Hajra’s reagent (0.2 M H_3_PO_4_, 1 M KCl) was added, mixed by vigorous shaking and centrifuged at 3000 rpm for 5 min and RT. After stratification, the lower layer of chloroform was extracted to another glass tube and nitrogen flow was blown to 200 μl. A phase (isopropyl alcohol:hexane:100 mm ammonium acetate at 58:40:2) was added to 1 ml. Finally, the sample was put into a centrifuge at 14,000 rpm at 4 °C for 20 min. We adopted normal phase liquid chromatography and coupled triple-quadrupole mass spectrometer (QTRAP^®^ 6500, SCIEX, Framingham, MA, USA) to extract the lipid for the positive and negative electrospray ionization mode, and Q-Trap in the multiple reaction monitoring mode operation to scan the precursor/product ion. Each experiment was repeated thrice. With multiple reaction monitoring data processing MultiQuant ™ software (AB SCIEX), the peak area of each pair was quantified [7]. The formic acid, acetone, acetonitrile, dithiothreitol, iodoacetamide, ammonium bicarbonate, and Tris base (Analytical Grade) were purchased from Sigma-Aldrich (St. Louis, MO, USA), the internal standard cocktails from Avanti Lipids Polar (MA, USA), and high-performance liquid chromatography (HPLC) grade chloroform (CHCl_3_), methanol, isopropyl alcohol (IPA), hexane, and ammonium acetate (NH_4_OAc) from Merck Millipore (Billerica, MA, USA).

### Measurement of lipidomic profiles

Lipid samples were derived through DB-23 column (30 m × 0.25 mm × 0.25 μm) (Agilent Technologies, Santa Clara, CA, USA) with high purity helium as a carrier gas with the flow rate of 1.5 ml/min. During the process, 2.0 μl was added, 50:1 s the split ratio, 220 °C as the ignition chamber temperature, and 150 °C as the injection port temperature. The temperature started from 150 °C and with an increasing program, reached 250 °C at the speed of 4 °C/min, and was maintained at 250 °C for 5 min. Working conditions of mass spectrometry mainly included the ionization source as EI, the ionization voltage at 70 eV, the ion source temperature at 200 °C, solvent delaying for 4 min, the multiplier voltage at 0.9 kV, and the scan range was 50–650 amu. and subjected to liquid chromatography-mass spectrometer analysis (FOCUS DSQTM II, Thermo Fisher Scientific).

Lipid extracts were loaded onto and then eluted with an Ultremex silica column (250 × 4.6 mm, 5 μm) and fitted with a 2 × 4 mm silica guard cartridge (Phenomenex, Torrance, CA, USA). The sample elation was performed as a 300 nl/min gradient, during which 50% B was from 0 to 5 min, ramped to 100% from 5 to 30 min linearly for 10 min, and returned 50% from 40 to 41 min until the end of the run at 50 min. The Q-Trap was operated in the multiple-reaction monitoring mode and the different precursor/product ion pairs with the mode, including PE (16:0/16:1):688.8–255.1, PE (16:0/17:1):702.8–255.1, PC (16:0/15:1):716.8–255.1, PE (18:1/18:1):742.8–281.1, PE (18:0/18:1):744.8–283.1, PS (18:1/18:0):788.6–281.1 (in the electrospray ionization-mode), Cer (d18:1/16:0):538.8–264.1, Cer (d18:1/24:0):650.8–264.1 (in the electrospray ionization + mode). Experiments of multiple-reaction monitoring mode for 502 lipids of plasma samples were also carried out to get lipids possible chemical structures and to scan pairs and quantification results. Data were processed with MultiQuant™ software (AB SCIEX) and the peak area of each pair was used for further quantification.

### Lipidomic data analysis

Multivariate statistical analysis and cluster analysis were performed using MetaboAnalyst 4.0 (http://www.metaboanalyst.ca). No lipids were filtered out based on 50% missing data criteria. Prior to analysis, missing values were replaced by median and pareto-scaling was used for normalization of all metabolomic MS intensity data. In addition, the Metaboanalyst software was used to make heat map of these three diseases and healthy people, dimensionality reduction, or the remaining five main indicators. The expression of more than 500 lipids was clearly shown.

### Statistical analysis

Data were presented as mean ± SE. Statistical significance of differences among groups were determined by one-way ANOVA test and the difference between each two groups by Student’s *T* test with one way and two tails. Fold changes of each elements in disease group above health control group were calculated on basis of the average in each group. Data obtained from the mass spectra were statistically analyzed using Simca, to obtain the picture of disease classification. Volcano maps of the data were based on patients with SAP, APE, or AECOPD, respectively. p value < 0.05 was considered to have statistical significance. A VIP plot was further used to rank the lipids based on their importance to differentiate the four groups. To capture the correlation between clinical phenomes and lipid elements, we used the lipid-QTL model which was modified from the eQTL-like effect model to estimate the association of clinical phenomes with lipid elements. To explore phenome-associated or specific lipid element changes, the values of lipid element quantitative trait loci (lQTL) matrixlQTL R package was applied to obtain the significant associated phenome-lipid element pairs and corresponding p-values, as shown in Additional file 1: Table S1–S3. MatrixlQTL implemented the linear model with both additive and dominant effects. We use Graph Pad Prism to make the ROC curve to measure the diagnostic accuracy/the value of early diagnosis between the specific-alternations of lipid elements with clinical phenomes in SAP, APE, or AECOPD.

## Results

DESS values of clinical phenomes in patients with SAP, APE, or AECOPD were listed in Additional file 1: Table S2, according to patient’s chief complaint, physician examination, biochemical analyses, and imaging. The sums of clinical phenomes were 63 ± 11, 30 ± 10, or 41 ± 22 in patients with SAP, APE, or AECOPD, respectively. In order to define the frequency and severity of clinical phenomes in diseases, we ranked the average of patient DESS values with significance as compared with the control and observed the appearance of each phenome among diseases in top 10. We found that d-dimer, expectoration, and tachypnea appeared in all groups, in addition to which cough, pulmonary nodule, pleural thickening, and erythrocyte sedimentation rate in SAP and AECOPD, C-reaction protein in SAP and APE, or heart rate in APE and AECOPD, as shown in Table 1. About 10% of total phenomes showed the statistical significance between the two groups, while no phenomes appeared significant among all three groups (Table 2). Of those, most also appeared in top 10 of clinical phenomes in Table 1. Of top 10 clinical phenomes, white blood cells and chest auscultation were altered in SAP, pulmonary embolism, blood sugar, diabetes, nutritional status, or prothrombin time in APE, or emphysema and chest distress in AECOPD.

**Table 1:** Top 10 clinical phenomes of patients with severe acute pneumonia (SAP), acute pulmonary embolism (APE), or acute exacerbation of chronic pulmonary diseases (AECPD) selected from the highest clinical phenotype score (mean ± SE)

SAP		APE		AECPD	
Cough	2.82 ± 0.13	d-dimer (mg/l)	2.88 ± 022	Cough	2.67 ± 0.16
Erythrocyte sedimentation rate	2.36 ± 0.16	Pulmonary embolism	2.30 ± 0.28	Expectoration	2.08 ± 0.15
Pulmonary nodule	2.18 ± 0.19	Blood sugar	1.45 ± 0.26	Pulmonary nodule	2.08 ± 0.17
White blood cells	2.18 ± 0.19	Prothrombin time	1.43 ± 0.27	Pleural thickening	1.67 ± 0.17
d-dimer	2.18 ± 0.19	Heart rate	1.17 ± 0.28	Tachypnea	1.42 ± 0.14
Expectoration	2.09 ± 0.13	Diabetes	1.16 ± 0.28	Heart rate	1.33 ± 0.16
Tachypnea	1.82 ± 0.17	C-reaction protein	1.15 ± 0.21	Erythrocyte sedimentation rate	1.33 ± 0.15
Pleural thickening	1.82 ± 0.19	Tachypnea	1.02 ± 0.22	Emphysema	1.33 ± 0.16
Chest auscultation	1.82 ± 0.19	Nutritional status	0.86 ± 0.04	Chest distress	1.17 ± 0.13
C-reaction protein	1.82 ± 0.14	Expectoration	0.74 ± 0.21	d-dimer	1.00 ± 0.15

**Table 2:** Clinical phenomes of patients with severe acute pneumonia (SAP), acute pulmonary embolism (APE), or acute exacerbation of chronic pulmonary diseases (AECPD) with statistical significance between each two groups

	APE vs SAP		AECPD vs SAP		AECPD vs APE	
	Fold	p value	Fold	p value	Fold	p value
Cough	0.21	0.01*	0.95	0.84	4.48	0.04*
d-dimer (mg/l)	1.32	0.19	0.46	0.16	0.35	0.01*
Wheeze	0.29	0.22	1	0.04*	3.29	0.27
Chest auscultation	0.01	0.04*	0.55	0.33	46.43	0.17
C-reaction protein	0.63	0.37	0.28	0.02*	0.43	0.23
Pulmonary embolism	6.32	0.00*	0.92	0.95	0.14	0.00*
Respiratory rate (times/min)	0.02	0.01*	0.41	0.25	24.25	0.46
Fever (℃)	0.49	0.43	0.14	0.04*	0.29	0.38
Prothrombin time (s)	2.62	0.24	0.00	0.13	0.00	0.02*
Performance status scores	0.35	0.30	0.00	0.03*	0.00	0.04*
Hemoglobin (g/l)	0.79	0.80	0.00	0.02*	0.00	0.20
Blood platelet (*10^9^)	0.00	0.08	0.00	0.02*		
Lung consolidation	0.00	0.13	0.00	0.04*		

Folds of digital evaluation score system (DESS) scores above SAP or APE were also listed

*The symptoms with statistical significance compared with the two groups of diseases (p<0.05)

**Fig. 2:**
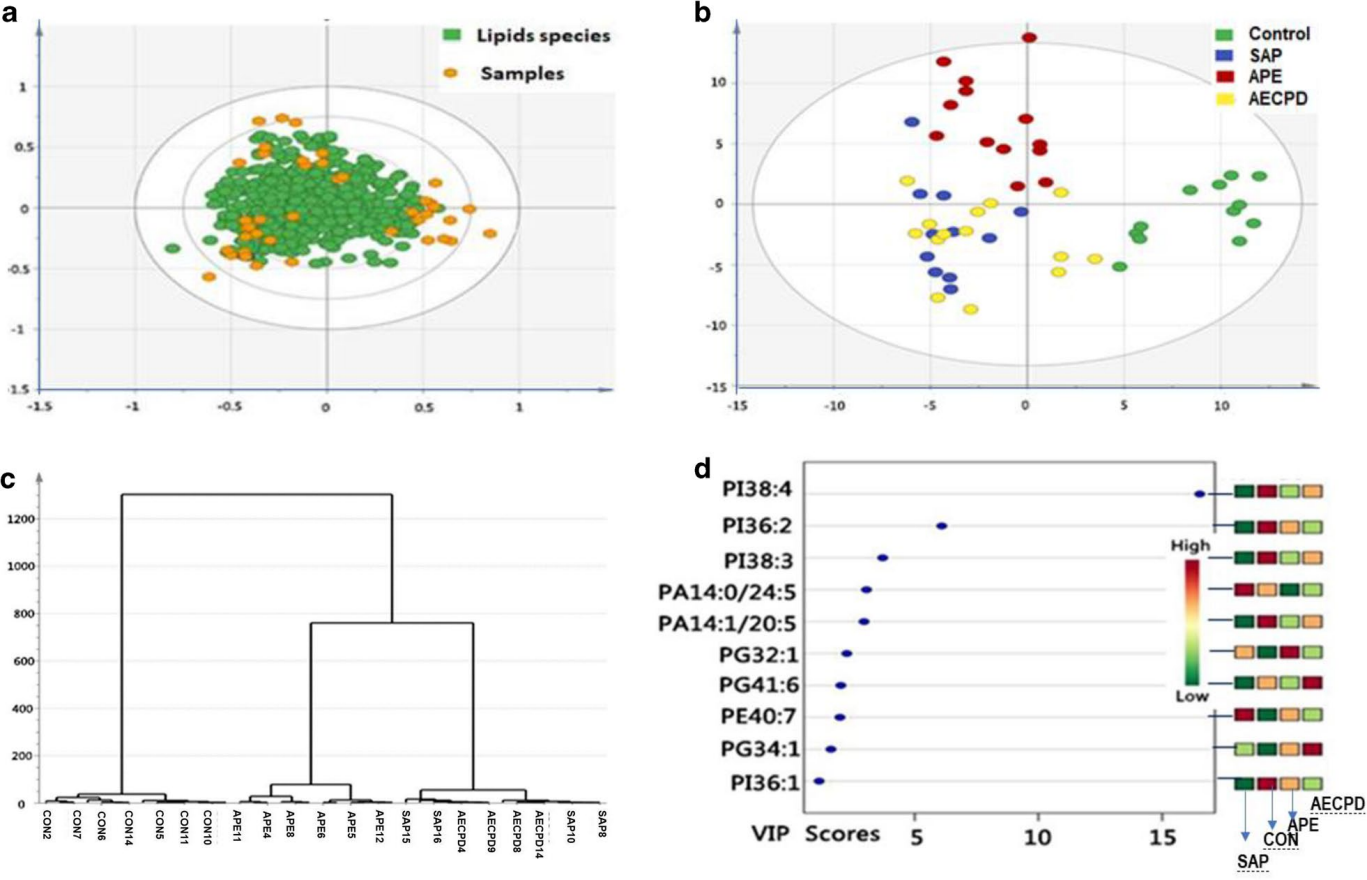
Lipid species and scatters performed with the orthogonal partial least squares discriminant analysis (**a**), where green dots represent 502 kinds of lipids species and yellow dots represent patient samples. The two dimensions of those lipids in healthy patients as control (CON), or patients with severe acute pneumonia (SAP), acute pulmonary embolism (APE), or acute exacerbation of chronic pulmonary diseases (AECOPD) (**b**) to represent the distribution of lipid elements. The hierarchical decomposition of lipid species, of which each cluster is the correlation of two adjacent samples where the distinction is better classified between CON and APE, while not but between SAP and AECOPD) (**c**). The VIP score map derived from the lipid elements to distinguish among top 10 contributions from each group of patients. Color from green to red indicates a low to high score, of which the higher score shows the greater contribution (**d**)

**Fig. 3:**
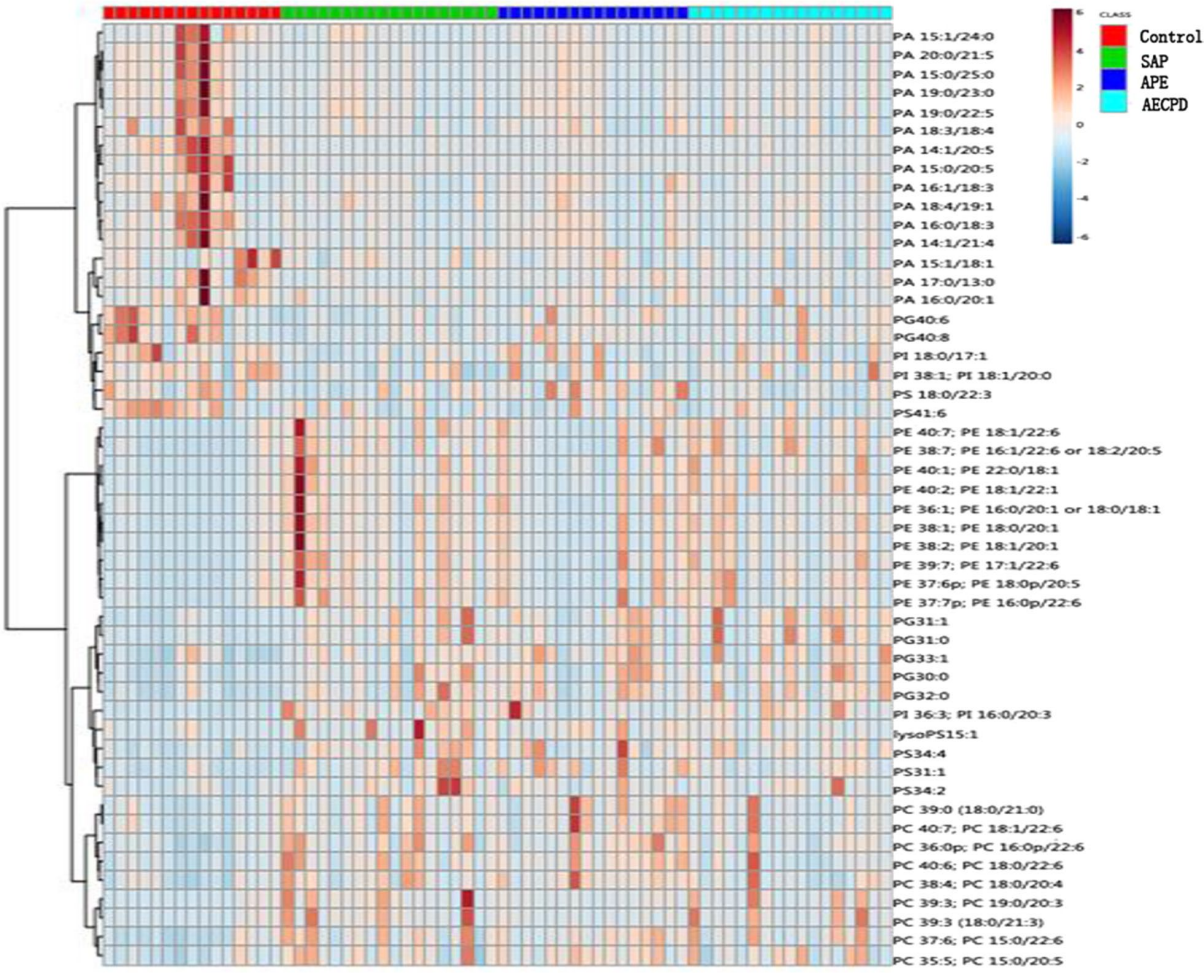
Map of top 50 lipid elements with the highest expression in patients with control, with severe acute pneumonia (SAP), acute pulmonary embolism (APE), or acute exacerbation of chronic pulmonary diseases (AECOPD)

The quality of lipidomic profiles measured and analyzed was presented in Fig. 1. Orthogonal Partial Least Squares Discriminant Analysis (OPLS-DA) maps made by SIMCA statistical software demonstrated the stronger correlation of 502 lipid elements among groups (Fig. 1a). Bi-dimension of 502 indicators and two plane projections of comprehensive analysis showed a clear distinguish among SAP, APE, and Healthy controls, while a difficult separation between SAP and AECOPD (Fig. 1b). The reliability of bi-dimensional projections was furthermore proved by clusters where patients were identified into subgroups and the same category of disease was aggregated (Fig. 1c). The VIP score chart with 10 lipid elements on the left represents the main lipid elements to distinguish among four groups, i.e. the contribution of 10 lipid elements from high to low in order of PI38:4, PI36:2, PI38:3, PA14:0/24:5, PA14:1/20:5, PF32:1, PG41:6, PE40:7, PG34:1, PI36:1, as shown in Fig. 1d. There is a clear correlation between lipid contributions and VIP score values. General values of 502 lipid elements of patients with control, SAP, APE, or AECOPD in the hot map demonstrate the difference among groups in Additional file 2: Figure S1A, B. Of those, about 50 of elements changed significantly as compared with control were mapped to show the clear difference among groups (Figs. 2, 3).

Table 3 demonstrates that more than twofold elevated lipid elements of patients with SAP, APE or AECOPD with statistical significance, as compared with healthy control (p < 0.05 or less). About 90 or 70 lipid elements in SAP or APE and AECOPD were significantly more than twofolds(Table 3) above levels of controls, respectively. In elevated elements, there are 30% phosphatidylethanolamine (PE), 20% lyso-lipids, 14% phosphatidylserine (PS), 12% phosphatidylglycerol (PG), and 11% phosphatidylcholine (PC) in SAP patients; 34% lyso-lipids, 18% PS, 15% PG, 13% PE, and 11% PC in APE; or 33% PE, 19% PG, 16% lyso-lipids, 13% PC, and 10% PS in AECOPD, respectively. While, there were about 20 elements in disease groups significantly less than twofolds below levels of controls, as shown in Table 4. Of declined those, 67% phosphatidic acids (PA), 19% PG, and 10% phosphatidylinositol (PI) in SAP; 64% PA, 21% PS, and 13% PG in APE; or 84% PA and 16% PS in AECOPD. There are 23 elements only specific elevated in SAP, 22 in APE, or 8 in AECOPD, respectively (Table 5), while 4–5 elements only specific declined in disease groups, as shown in Table 5. There were 33 or 9 common elements elevated or declined significantly in all SAP, APE, and AECOPD, as compared with healthy control (p < 0.05 or less, respectively, Table 6).

**Table 3:** More than twofold elevated lipid elements of patients with severe acute pneumonia (SAP), acute pulmonary embolism (APE), or acute exacerbation of chronic pulmonary diseases (AECPD) with statistical significance, as compared with healthy control (p < 0.05 or less)

SAP			APE			AECPD		
Elements	Folds	p_value_	Elements	Folds	p_value_	Elements	Folds	p_value_
PC 18:1/23:1	25.49	0.05	PS30:1	8.12	0.00	PC 18:1/23:1	12.39	0.01
d181So	21.71	0.04	PI 36:3	6.86	0.02	PI 36:3	8.21	0.00
PS30:1	12.38	0.02	PS31:1	5.63	0.00	PS30:1	7.21	0.00
PI 36:3	9.59	0.00	lysoPE19:0	5.09	0.05	lysoPE19:0	6.92	0.03
lysoPS17:1	8.70	0.03	PG30:1	4.89	0.00	lysoPI 22:4 (sn-1)	5.55	0.03
lysoPE19:0	8.66	0.02	PS31:0	4.70	0.00	PC 41:6	5.50	0.03
lysoPI22:6 (sn-1)	7.41	0.01	lysoPS18:2	4.15	0.01	PG30:1	5.26	0.00
PE 40:3	6.96	0.02	PC 16:0/26:0	3.98	0.02	PG31:0	5.21	0.00
PS30:0	6.74	0.03	lysoPC22:0 (sn-1)	3.70	0.04	PE 40:3	5.06	0.00
PE 40:2	6.56	0.00	PE 40:3	3.68	0.01	PE 40:2	4.95	0.00
PC 41:6	6.56	0.03	PS30:0	3.60	0.05	PG31:1	4.86	0.00
PE 40:1	6.19	0.00	PE 40:2	3.52	0.01	lysoPI22:6 (sn-1)	4.83	0.00
PE 42:8	6.02	0.03	PS37:6	3.51	0.03	PE 40:1	4.76	0.00
lysoPE22:6 (sn-1)	5.99	0.05	PE 40:1	3.50	0.01	PS30:0	4.18	0.00
lysoPG14:0	5.90	0.01	PG30:0	3.50	0.00	PE 38:7	4.09	0.00
PE 38:1	5.76	0.00	PG31:0	3.49	0.00	PC 39:4 (18:0/21:4)	4.00	0.03
lysoPS16:0	5.44	0.01	PG31:1	3.47	0.00	lysoPC 15:1 (sn-1)	4.00	0.04
lysoPS15:1	5.44	0.01	lysoPS15:1	3.42	0.01	PC 39:3 (18:0/21:3)	3.95	0.03
PE 39:7	5.33	0.00	PE 39:7	3.27	0.02	PE 38:1	3.90	0.00
PS31:1	5.32	0.00	PC 19:0/21:2	3.24	0.02	PE 40:7	3.82	0.00
PE 38:2	5.22	0.00	PE 42:8	3.13	0.04	PE 36:5	3.67	0.00
PE 40:7	4.86	0.00	lysoPS16:1	3.09	0.01	PE 39:7	3.61	0.00
PG30:1	4.78	0.00	PE 38:1	3.06	0.04	lysoPE19:1	3.61	0.04
PE 38:3	4.60	0.02	PC 19:0/19:0	3.05	0.02	PE 42:8	3.58	0.01
PC39:3 (18:0/21:3)	4.58	0.01	PE 38:7	3.01	0.05	lysoPS18:2	3.44	0.00
PC 39:4 (18:0/21:4)	4.57	0.01	lysoPS17:0	3.01	0.01	PC 39:3	3.39	0.02
PC 39:3	4.46	0.01	lysoPC15:1 (sn-1)	2.87	0.01	PG30:0	3.39	0.00
PS32:0	4.43	0.03	PG34:0	2.84	0.01	PE 38:5	3.33	0.00
PE 40:4	4.30	0.04	PG34:1	2.80	0.01	PE 38:2	3.29	0.01
PG31:0	4.28	0.00	lysoPS19:0	2.74	0.01	lysoPS22:6	3.28	0.05
PE 38:7	4.25	0.00	PG32:0	2.65	0.00	lysoPC 17:1 (sn-1)	3.24	0.04
PG31:1	4.19	0.00	PE 38:3	2.60	0.03	PC 19:0/21:2	3.24	0.03
PE 38:5	4.18	0.01	lysoPC22:6 (sn-1)	2.56	0.01	PC 39:5 (18:0/21:5)	3.20	0.03
PE 36:1	4.10	0.00	lysoPG16:1	2.55	0.04	PS31:1	3.17	0.01
PS32:1	4.07	0.01	PS34:4	2.50	0.02	PE 38:3	3.06	0.01
lysoPI20:2 (sn-1)	4.02	0.02	lysoPC19:0 (sn-1)	2.50	0.01	PE 40:4	3.03	0.02
PE 36:5	3.99	0.00	PG32:2	2.49	0.01	PE 37:6p	3.00	0.01
PE 37:7p	3.97	0.00	lysoPC20:3 (sn-1)	2.48	0.02	PG34:0	2.97	0.00
PE 37:6p	3.83	0.00	PS34:5	2.43	0.03	PG32:0	2.96	0.00
lysoPS18:2	3.83	0.01	lysoPC20:5 (sn-1)	2.40	0.01	PC 39:6	2.87	0.02
PE 35:6p	3.80	0.01	lysoPC16:1 (sn-1)	2.40	0.01	PS31:0	2.85	0.01
PC 39:5 (18:0/21:5)	3.77	0.01	PG32:1	2.40	0.01	PG34:1	2.84	0.01
lysoPS14:0	3.64	0.02	PE 35:5p	2.40	0.02	PG34:5	2.81	0.00
lysoPI19:0 (sn-1)	3.52	0.00	PC39:0 (18:0/21:0)	2.36	0.00	lysoPI20:0 (sn-1)	2.80	0.03
PC 39:6	3.51	0.01	PS34:3	2.35	0.05	PE 35:5p	2.73	0.01
PC39:2 (18:0/21:2)	3.45	0.02	lysoPC18:0 (sn-1)	2.33	0.01	PE 35:6p	2.72	0.01
lysoPE19:1	3.34	0.03	lysoPC20:4 (sn-1)	2.31	0.01	lysoPS20:4	2.71	0.03
lysoPS18:3	3.32	0.01	lysoPC18:3 (sn-1)	2.30	0.03	PE 38:6	2.64	0.00
PG30:0	3.31	0.00	PC 41:6	2.30	0.00	PG32:1	2.52	0.01
PE 40:6	3.22	0.01	PG33:1	2.28	0.00	PE 37:7p	2.51	0.03
PE 35:5p	3.15	0.00	PS36:6	2.27	0.04	PE 36:6	2.48	0.01
PC 19:0/21:2	3.15	0.02	lysoPC20:2 (sn-1)	2.26	0.03	PG32:2	2.47	0.01
PS35:3	3.12	0.01	PE 35:6p	2.25	0.04	lysoPG15:0	2.46	0.04
PC 19:0/19:0	2.99	0.01	lysoPC17:0 (sn-1)	2.22	0.01	PE 40:6	2.44	0.01
PG34:0	2.97	0.02	PC 40:6	2.20	0.00	PS35:3	2.40	0.03
PS34:2	2.94	0.01	PS35:3	2.20	0.02	lysoPC16:1 (sn-1)	2.37	0.04
PE 42:7	2.92	0.04	lysoPC20:0 (sn-1)	2.20	0.03	PS34:3	2.35	0.00
PG32:0	2.92	0.00	lysoPS20:5	2.20	0.03	PG34:4	2.32	0.01
PS37:6	2.89	0.01	PS38:7	2.18	0.01	PS37:6	2.27	0.00
PE 37:5p	2.87	0.02	PC 40:7	2.17	0.00	PE 36:4	2.22	0.01
lysoPC 22:0 (sn-1)	2.80	0.01	PS36:5	2.14	0.05	PG33:0	2.22	0.02
lysoPS19:0	2.77	0.01	lysoPC16:0 (sn-2)	2.12	0.01	PS34:4	2.22	0.00
PG32:2	2.71	0.00	lysoPC15:0 (sn-2)	2.12	0.04	PE 36:1	2.20	0.03
PS34:3	2.68	0.01	PS33:0	2.11	0.02	PE 37:5p	2.20	0.03
PG34:1	2.68	0.00	PC 39:4 (18:0/21:4)	2.09	0.00	PG33:1	2.18	0.01
PS34:4	2.67	0.00	lysoPC 18:1 (sn-1)	2.05	0.02	PC 39:7	2.13	0.01
PE 38:6	2.67	0.01	PG33:0	2.04	0.05	PS37:5	2.10	0.01
PE 37:3	2.66	0.05	PC 40:4	2.00	0.00	PC 37:3	2.09	0.04
PS36:5	2.64	0.01				PG36:2	2.08	0.01
PE 36:6	2.60	0.01				PE 38:4	2.08	0.02
PS34:5	2.58	0.00						
PE 38:4	2.53	0.02						
PC 39:7	2.49	0.01						
PG34:5	2.48	0.01						
PG34:4	2.46	0.00						
PC 37:4	2.44	0.02						
lysoPG16:1	2.43	0.01						
PG32:1	2.36	0.00						
PC 37:3	2.35	0.02						
PE 36:4	2.30	0.01						
PE 35:1	2.26	0.04						
PS35:2	2.23	0.01						
PS33:2	2.23	0.04						
PC 40:4	2.19	0.00						
PG33:0	2.11	0.02						
PE 39:6	2.11	0.02						
PC 40:6	2.11	0.00						
lysoPG18:3	2.08	0.04						
PC 40:5	2.06	0.00						
PC 40:8	2.01	0.03

**Table 4:** Less than twofold declined regulated lipid elements of patients with severe acute pneumonia (SAP), acute pulmonary embolism (APE), or acute exacerbation of chronic pulmonary diseases (AECPD) with statistical significance, as compared with healthy control (p < 0.05 or less)

SAP			APE			AECPD		
Elements	Folds	p_value_	Elements	Folds	p_value_	Elements	Folds	p_value_
PS41:6	0.50	0.01	PA 16:0/20:1	0.50	0.03	PA 16:0/20:1	0.47	0.02
PA 16:0/20:1	0.48	0.02	PS41:6	0.49	0.02	PS41:6	0.46	0.01
PI 38:1	0.47	0.00	PA 16:0/18:3	0.48	0.04	PS 18:0/22:3	0.46	0.01
PG40:5	0.47	0.03	PG39:3	0.43	0.05	PS 20:3/22:6	0.45	0.02
PA 18:3/18:4	0.43	0.01	PA 17:0/13:0	0.41	0.04	PA 10:0/18:1	0.44	0.02
PG39:3	0.41	0.03	PA 15:1/24:0	0.39	0.03	PA 10:0/18:0	0.43	0.00
PG37:1	0.40	0.04	PS38:1	0.39	0.02	PA 18:3/18:4	0.41	0.02
PI 18:0/17:1	0.39	0.00	PG37:1	0.36	0.04	PA 16:1/18:3	0.38	0.02
PA 16:1/18:3	0.38	0.02	PA 18:0/20:5	0.35	0.02	PA 15:1/24:0	0.37	0.02
PA 18:4/19:1	0.38	0.04	PS40:1	0.32	0.04	PA 14:1/21:4	0.36	0.03
PA 19:0/22:5	0.37	0.04	PA 20:0/21:5	0.31	0.02	PA 18:4/19:1	0.35	0.03
PG40:6	0.33	0.00	PA 15:0/25:0	0.29	0.04	PA 19:0/22:5	0.33	0.03
PA 15:1/24:0	0.33	0.01	PA 14:0/24:5	0.18	0.04	PA 16:1/18:4	0.33	0.03
PA 14:1/21:4	0.32	0.02	PA 14:1/20:5	0.11	0.00	PA 16:0/18:3	0.32	0.01
PG40:8	0.31	0.00	PA 15:0/20:5	0.04	0.01	PA 17:0/13:0	0.30	0.01
PA 16:0/18:3	0.31	0.00				PA 19:0/23:0	0.29	0.03
PA 19:0/23:0	0.31	0.03				PA 20:0/21:5	0.25	0.01
PA 17:0/13:0	0.28	0.01				PA 15:0/25:0	0.22	0.02
PA 18:1/20:4	0.25	0.04				PA 14:1/20:5	0.10	0.00
PA 20:0/21:5	0.22	0.01				PA 15:0/20:5	0.06	0.00
PA 15:0/25:0	0.20	0.01						
PA 14:1/20:5	0.08	0.00						
PA 15:0/20:5	0.04	0.00

**Table 5:** Disease-specific profiles of elevated twofold lipid elements of patients with severe acute pneumonia (SAP), acute pulmonary embolism (APE), or acute exacerbation of chronic pulmonary diseases (AECPD) with statistical significance, as compared with healthy control (p < 0.05 or less)

SAP			APE			AECPD		
Elements	Folds	p_value_	Elements	Folds	p_value_	Elements	Folds	p_value_
Elevated twofold lipid elements								
d181So	21.71	0.04	PC 16:0/26:0	3.98	0.02	lysoPI 22:4 (sn-1)	5.55	0.03
lysoPS17:1	8.70	0.03	lysoPS16:1	3.09	0.01	lysoPS22:6	3.28	0.05
lysoPE 22:6 (sn-1)	5.99	0.05	lysoPS17:0	3.01	0.01	lysoPC 17:1 (sn-1)	3.24	0.04
lysoPG14:0	5.90	0.01	lysoPC 22:6 (sn-1)	2.56	0.01	lysoPI 20:0 (sn-1)	2.80	0.03
lysoPS16:0	5.44	0.01	lysoPC 19:0 (sn-1)	2.50	0.01	lysoPS20:4	2.71	0.03
PS32:0	4.43	0.03	lysoPC 20:3 (sn-1)	2.48	0.02	lysoPG15:0	2.46	0.04
PS32:1	4.07	0.01	lysoPC 20:5 (sn-1)	2.40	0.01	PS37:5	2.10	0.01
lysoPI 20:2 (sn-1)	4.02	0.02	PC 39:0	2.36	0.00	PG36:2	2.08	0.01
lysoPS14:0	3.64	0.02	lysoPC 18:0 (sn-1)	2.33	0.01			
lysoPI 19:0 (sn-1)	3.52	0.00	lysoPC 20:4 (sn-1)	2.31	0.01			
PC 39:2	3.45	0.02	lysoPC 18:3 (sn-1)	2.30	0.03			
lysoPS18:3	3.32	0.01	PS36:6	2.27	0.04			
PS34:2	2.94	0.01	lysoPC 20:2 (sn-1)	2.26	0.03			
PE 42:7	2.92	0.04	lysoPC 17:0 (sn-1)	2.22	0.01			
PE 37:3	2.66	0.05	lysoPC 20:0 (sn-1)	2.20	0.03			
PC 37:4	2.44	0.02	lysoPS20:5	2.20	0.03			
PE 35:1	2.26	0.04	PS38:7	2.18	0.01			
PS33:2	2.23	0.04	PC 40:7	2.17	0.00			
PS35:2	2.23	0.01	lysoPC 15:0 (sn-2)	2.12	0.04			
PE 39:6	2.11	0.02	lysoPC 16:0 (sn-2)	2.12	0.01			
lysoPG18:3	2.08	0.04	PS33:0	2.11	0.02			
PC 40:5	2.06	0.00	lysoPC 18:1 (sn-1)	2.05	0.02			
PC 40:8	2.01	0.03						
Declined twofold lipid elements								
PA 18:1/20:4	0.25	0.04	PA 14:0/24:5	0.18	0.04	PA 10:0/18:0	0.43	0.00
PG40:5	0.47	0.03	PA 18:0/20:5	0.35	0.02	PA 10:0/18:1	0.44	0.02
PG40:6	0.33	0.00	PS38:1	0.39	0.02	PA 16:1/18:4	0.33	0.03
PG40:8	0.31	0.00	PS40:1	0.32	0.04	PS 18:0/22:3	0.46	0.01
PI 18:0/17:1	0.39	0.00				PS 20:3/22:6	0.45	0.02

**Table 6:** Common profiles of elevated twofold lipid elements of ALL patients with severe acute pneumonia (SAP), acute pulmonary embolism (APE), or acute exacerbation of chronic pulmonary diseases (AECPD) with statistical significance, as compared with healthy control (p < 0.05 or less)

Elements	SAP		APE		AECPD	
	Folds	p_value_	Folds	p_value_	Folds	p_value_
lysoPE19:0	8.66	0.02	5.09	0.05	6.92	0.03
lysoPS18:2	3.83	0.01	4.15	0.01	3.44	0.00
PC 19:0/21:2	3.15	0.02	3.24	0.02	3.24	0.03
PC 39:4	4.57	0.01	2.09	0.00	4.00	0.03
PC 41:6	6.56	0.03	2.30	0.00	5.50	0.03
PE 35:5p	3.15	0.00	2.40	0.02	2.73	0.01
PE 35:6p	3.80	0.01	2.25	0.04	2.72	0.01
PE 38:1	5.76	0.00	3.06	0.04	3.90	0.00
PE 38:3	4.60	0.02	2.60	0.03	3.06	0.01
PE 38:7	4.25	0.00	3.01	0.05	4.09	0.00
PE 39:7	5.33	0.00	3.27	0.02	3.61	0.00
PE 40:1	6.19	0.00	3.50	0.01	4.76	0.00
PE 40:2	6.56	0.00	3.52	0.01	4.95	0.00
PE 40:3	6.96	0.02	3.68	0.01	5.06	0.00
PE 42:8	6.02	0.03	3.13	0.04	3.58	0.01
PG30:0	3.31	0.00	3.50	0.00	3.39	0.00
PG30:1	4.78	0.00	4.89	0.00	5.26	0.00
PG31:0	4.28	0.00	3.49	0.00	5.21	0.00
PG31:1	4.19	0.00	3.47	0.00	4.86	0.00
PG32:0	2.92	0.00	2.65	0.00	2.96	0.00
PG32:1	2.36	0.00	2.40	0.01	2.52	0.01
PG32:2	2.71	0.00	2.49	0.01	2.47	0.01
PG33:0	2.11	0.02	2.04	0.05	2.22	0.02
PG34:0	2.97	0.02	2.84	0.01	2.97	0.00
PG34:1	2.68	0.00	2.80	0.01	2.84	0.01
PI 36:3	9.59	0.00	6.86	0.02	8.21	0.00
PS30:0	6.74	0.03	3.60	0.05	4.18	0.00
PS30:1	12.38	0.02	8.12	0.00	7.21	0.00
PS31:1	5.32	0.00	5.63	0.00	3.17	0.01
PS34:3	2.68	0.01	2.35	0.05	2.35	0.00
PS34:4	2.67	0.00	2.50	0.02	2.22	0.00
PS35:3	3.12	0.01	2.20	0.02	2.40	0.03
PS37:6	2.89	0.01	3.51	0.03	2.27	0.00
PA 14:1/20:5	0.08	0.00	0.11	0.00	0.10	0.00
PA 15:0/20:5	0.04	0.00	0.04	0.01	0.06	0.00
PA 15:0/25:0	0.20	0.01	0.29	0.04	0.22	0.02
PA 15:1/24:0	0.33	0.01	0.39	0.03	0.37	0.02
PA 16:0/18:3	0.31	0.00	0.48	0.04	0.32	0.01
PA 16:0/20:1	0.48	0.02	0.50	0.03	0.47	0.02
PA 17:0/13:0	0.28	0.01	0.41	0.04	0.30	0.01
PA 20:0/21:5	0.22	0.01	0.31	0.02	0.25	0.01
PS41:6	0.50	0.01	0.49	0.02	0.46	0.01

**Fig. 4:**
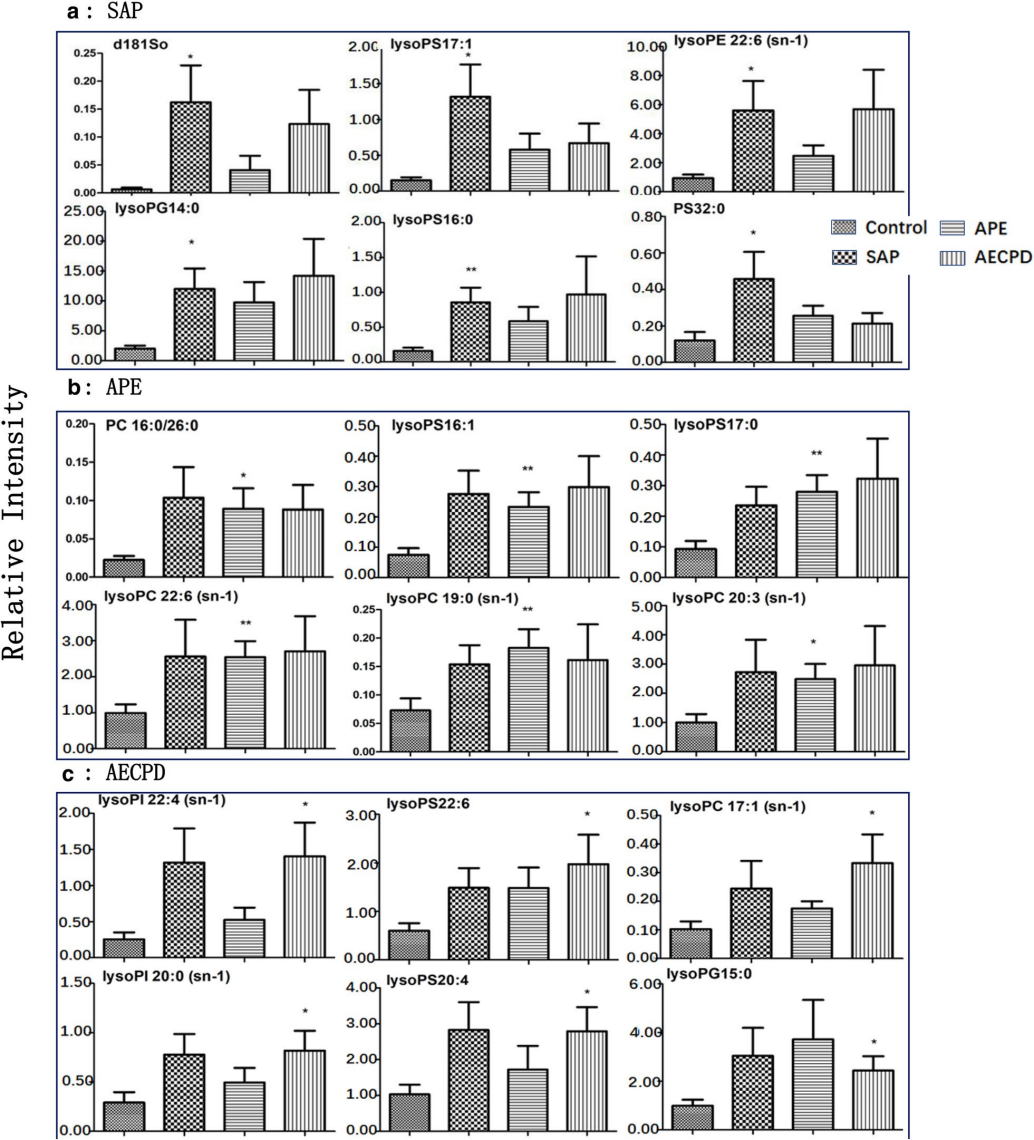
Top 6 lipid elements with the highest expression in patients with severe acute pneumonia (**a** SAP), acute pulmonary embolism (**b** APE), or acute exacerbation of chronic pulmonary diseases (**c** AECOPD), after comparing with healthy patients.*, **p values less than 0.05 and 0.01, respectively, as compared with healthy patients

**Fig. 5:**
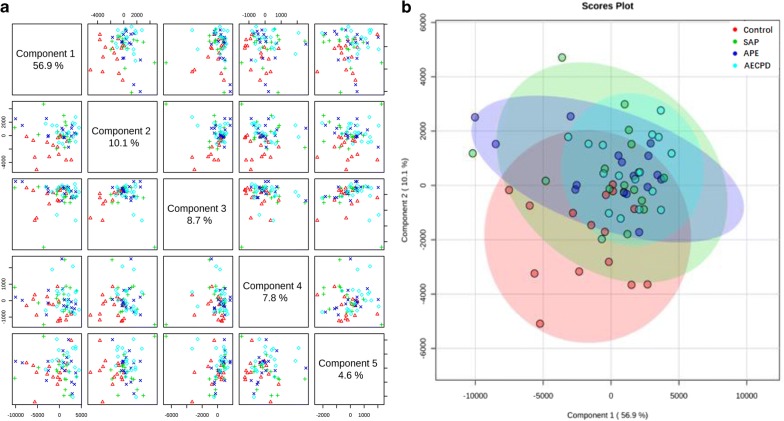
The analytic graph of five principal components as five dimensions of lipid elements selected from 502 dimensions (**a**). Two-dimensional plans (**b**) made by PLS-DA of the first two principal components PC1 as abscissa and PC2 as ordinate where healthy patients, severe acute pneumonia (SAP), acute pulmonary embolism (APE), or acute exacerbation of chronic pulmonary diseases (AECOPD) were clearly distinguished

**Fig. 6:**
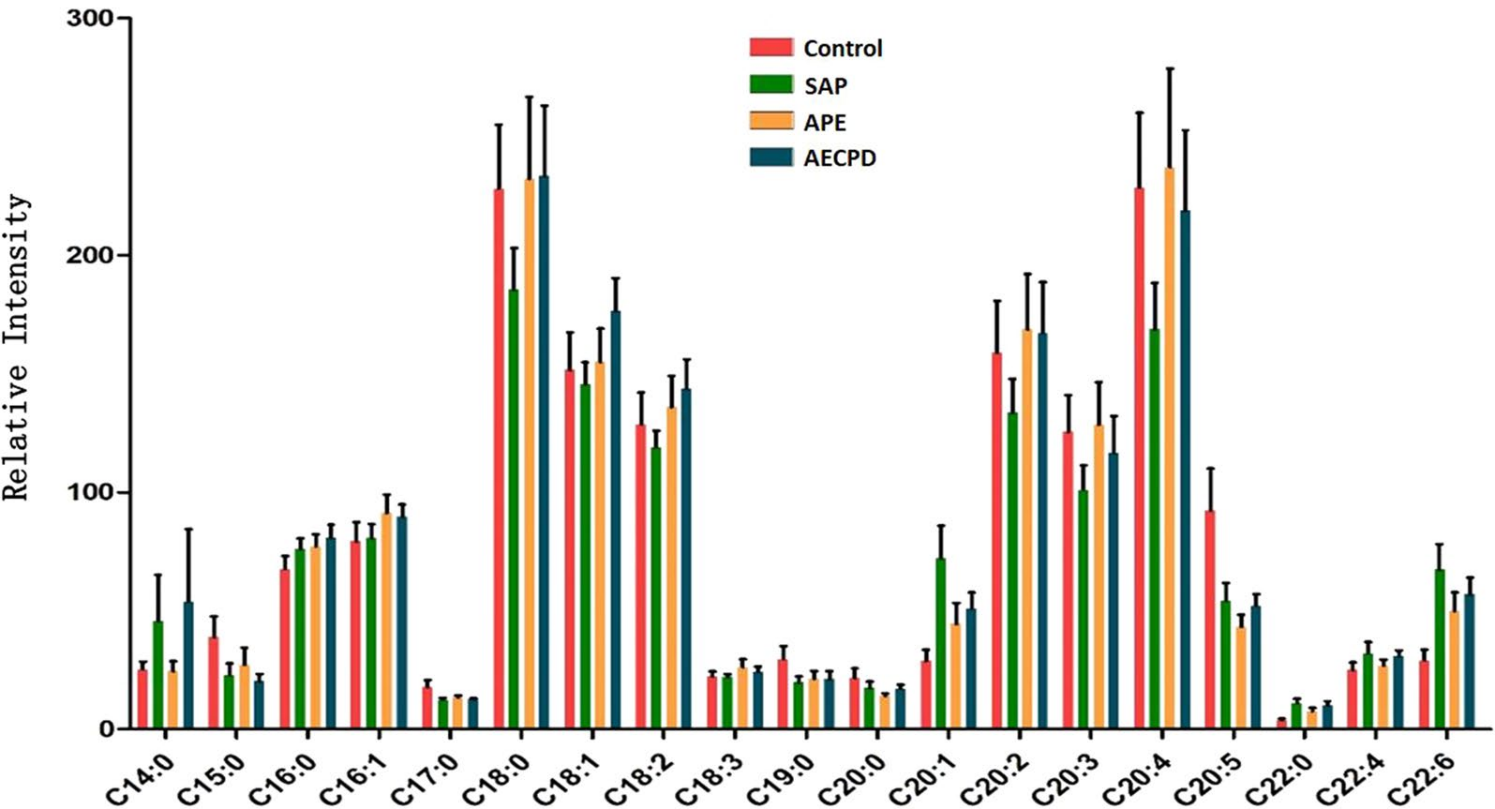
C-atom map based on the number of major C-atoms expressed by lipid species in healthy patients or patients with severe acute pneumonia (SAP), acute pulmonary embolism (APE), or acute exacerbation of chronic pulmonary diseases (AECOPD). Each column represents the mean ± SD of independent groups

**Fig. 7:**
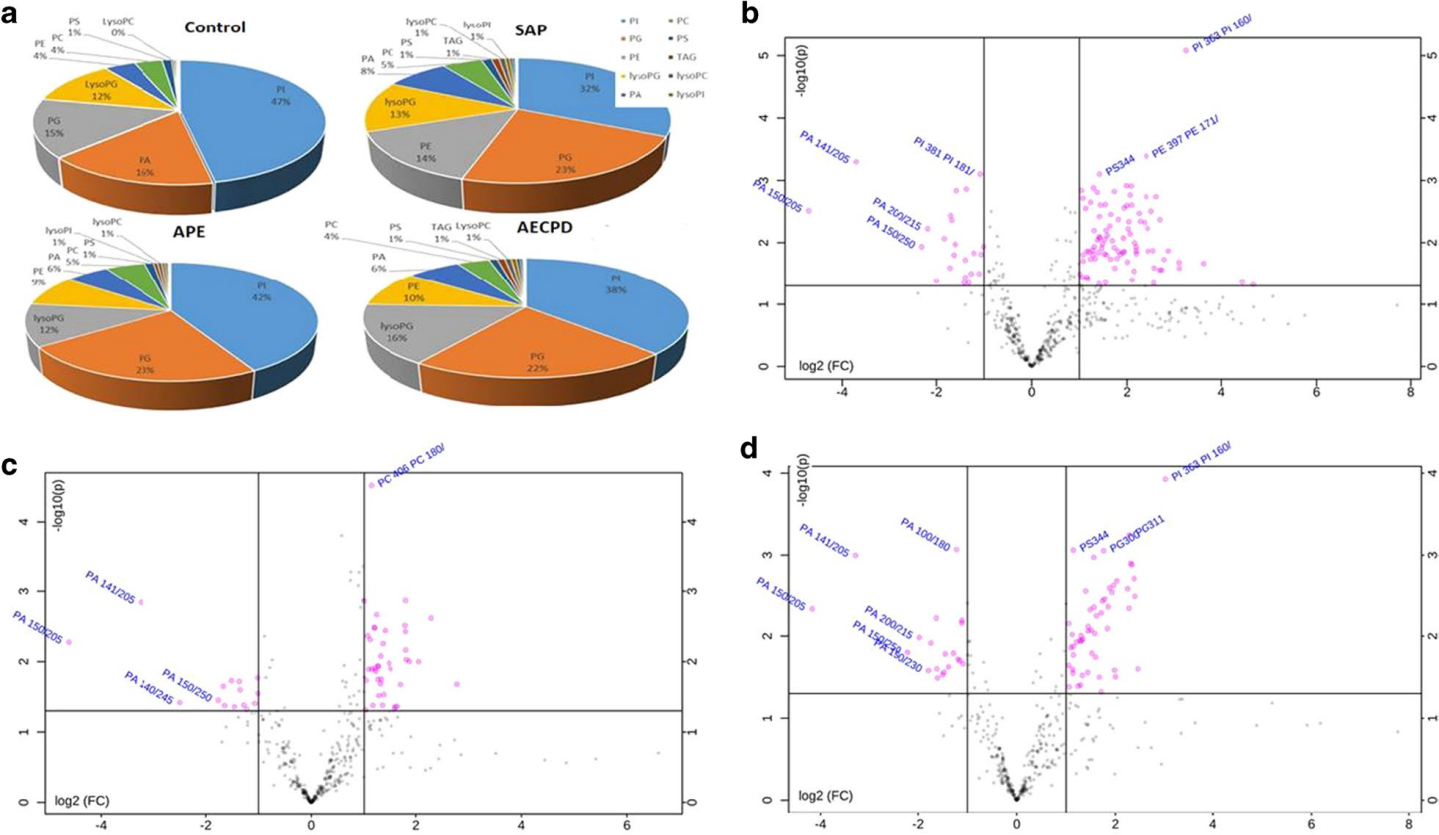
The proportion of main lipid components (**a**) in healthy patients or patients with severe acute pneumonia (SAP), acute pulmonary embolism (APE), or acute exacerbation of chronic pulmonary diseases (AECOPD). Volcano Plot with the transverse axis of the log2 (FC) and the longitudinal axis of − log 10 (p value) where every dot represents one lipid species of patients with severe acute pneumonia (**b**), acute pulmonary embolism (**c**), or acute exacerbation of chronic pulmonary diseases (**d**). The point outside the horizontal axis 1 means that the lipid element is more than twofolds higher than control group, whereas the one on the left side of − 1 is down-regulated by twofolds of lipid species. A line perpendicular to the Y axis is − log 10 (0.05), and points above this line indicate lipid species with significant difference < 0.05

The number of lipid elements elevated in all groups accounted for 30% of those in SAP and about 50% in APE and AECOPD, while declined numbers accounted for about 45% in disease groups. We selected the top 6 elements of the highest elevation in each group and found that most of them were also elevated in multiple groups, by comparing levels of the same elements from one group with those from others, as shown in Additional file 2: Figure S2A for SAP, 2B for APE, or 2C for AECOPD. Of those, the number of lipid elements that either elevated or declined significantly were listed in Table 5, we also selected the top 6 specific elements of the highest elevation from SAP (Fig. 4a), APE (Fig. 4b), or AECOPD (Fig. 4c). We were able to not only screen out the specific lipid elements of each group by comparing each group of diseases with those of healthy control, but also screened for statistically significant lipid elements for each group of diseases. The first three results with the highest up-regulation were shown in Table 7. The principal component analysis also demonstrated clear possibility to distinguish between healthy control and diseases, e.g. principal components 1, 2, 3, 4, and 5 were 56.9, 10.1, 8.7, 7.8, and 4.6% (Fig. 5a). Furthermore, the definite distribution of individual elements within dominate principal components 1 and 2 was observed between control and diseases and among other diseases (Fig. 5b). Both were accounted for more than 70% in the two-dimensional plan made by PLS-DA. We furthermore analyzed alterations of major C-atoms expressed within lipid elements and found that the major portions of C-atom elements were C18:0–C18:2 and C20:2–C20:4, respectively (Fig. 6). Levels of C14:0, C20:1, C22:4, or C22:6 were elevated and C18:0, C20:2, C20:3, or C20:4 were significantly declined in SAP, while C15:0, C17:0, or C20:5 levels declined and C20:1 or C22:6 were elevated in all SAP, APE, and AECOPD, respectively. Figure 7a showed a definite difference of major lipid distributions among groups which included 502 lipid elements, of which some were significantly elevated or declined in SAP (Fig. 7b), APE (Fig. 7c), or AECOPD (Fig. 7d).

**Table 7:** Top 3 more than twofold elevated lipid elements of patients with severe acute pneumonia (SAP), acute pulmonary embolism (APE), or acute exacerbation of chronic pulmonary diseases (AECPD) with statistical significance, as compared with each other (p < 0.05 or less)

**APE**		p		Fold	
Compared with SAP					
PG40:1		0.03		2.02	
PG40:6		0.01		2.04	
PS 18:0/22:3		0.01		2.07	

SAP	p	Fold	AECPD	p	Fold
Compared with APE					
PC 39:3(18:0/21:3)	0.03	2.79	lysoPG20:0	0.01	2.65
PC 39:3(19:0/20:3)	0.03	2.55			

SAP	p	Fold	APE	p	Fold
Compared with AECPD					
lysoPS15:1	0.05	2.53	PS 18:0/22:3	0.00	2.43
			PS 19:0/19:0	0.02	2.33
			PS 20:3/22:6	0.04	2.13

**Fig. 8:**
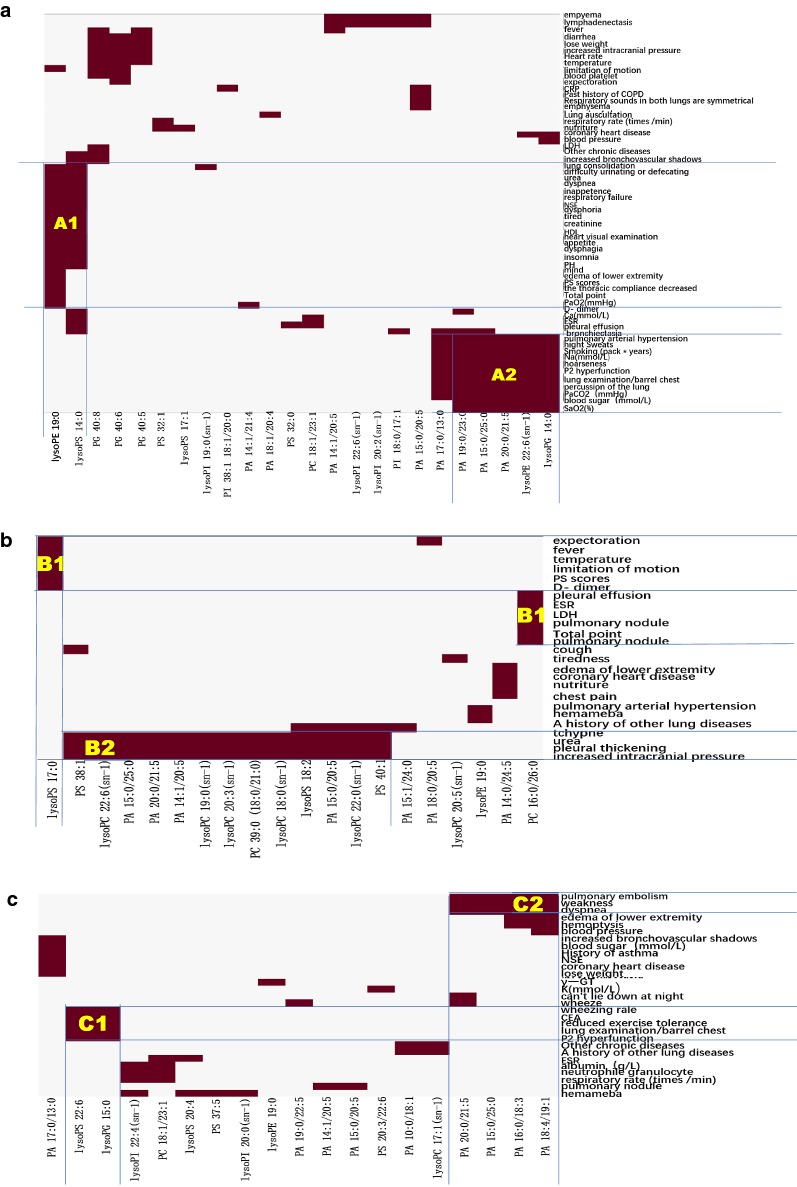
The specific correlation between clinical phenotypes and altered lipid elements of patients with severe acute pneumonia (**a**), acute pulmonary embolism (**b**), or acute exacerbation of chronic pulmonary diseases (**c**)

With the aim to identify disease phenome-specific lipid elements, circulating levels of lipid elements were integrated with clinical phenomes in patients with SAP (Fig. 8a), APE (Fig. 8b), or AECOPD (Fig. 8c), respectively. About 24, 20, or 19 lipid elements respectively, from those phenome-lipid element pairs of patients with SAP, APE, or AECOPD, had significant differences as shown in heatmap patterns. Various lipid elements and the corresponding clinical phenotype were clearly seen in Fig. 8, where, lysoPE 19:0 and lysoPS 14:0 were corresponded to 23 clinical phenotypes in SAP (Figure 8A1), rather than in APE and AECOPD. In addition, up-regulated lysoPG14:0 and lysoPE 22:6 or down-regulated PA 19:0/23:0 and PA 15:0/25:0 as well as PA 20:0/21:5 were corresponded to pulmonary hypertension, high sweats, smoking time, Na^+^, P2 hyperfunction, PaCO_2_, blood sugar, and SaO_2_ (Fig. 8A2). In patient with APE, up-regulated lysoPS17:0 and PC16:0/26:0 were highly correlated with clinical phenotypes (Fig. 8B1) andhad the highest specificity of disease, and differed from other diseases. Clinical phenomes, e.g. tchypne, urea, pleural thickening, and increased intracranial pressure, were associated with PS 38:1, lysoPC 22:6/18:0/22:0, PA 15:00/25:0/20:0/21:5/14:1/20:5/15:0/20:5, PC 39:0, lysoPS 18:2, PS 40:1 (Fig. 8B2). In AECOPD, LypoPS 22:6 and lysoPG 15:0 were highly correlated with wheezing rate, CEA, reduced exercise tolerance, lung examination/barrel chest, and P2 hyperfunction (Fig. 8C1), while PA 20:0/21:5/15:0/25:0/16:0/18:3/18:4/19:1 with pulmonary embolism, weakness, dypnea (Fig. 8C2). Additional file 2: Figures S3 and S6 demonstrated that diagnostic accuracies of specific lipid elements were 0.70–0.90, 0.70–0.80, or 0.70–0.90 in SAP, APE, or AECOPD, respectively (p < 0.05 or less). We screened out specific-increased lipid elements listed in Table 5, and listed the AUC levels of lipid element specificity with significant difference in Table 8.

**Table 8:** AUC values of specific lipid molecules in SAP, APE, AECPD, “*” indicated statistically significant

SAP		APE		AECPD	
Specific molecules	AUC	Specific molecules	AUC	Specific molecules	AUC
d181so	0.76*	LysoPC15:0(sn-2)	0.72*	LysoPC17:1(sn-1)	0.71*
LysoPE22:6(sn-1)	0.74*	LysoPC16:0(sn-2)	0.78*	LysoPG15:0	0.67
LysoPG14:0	0.85*	LysoPC17:0(sn-1)	0.77*	LysoPI20:0(sn-1)	0.74*
LysoPG18:3	0.70	LysoPC18:0(sn-1)	0.78*	LysoPI22:4(sn-1)	0.81*
LysoPI19:0	0.82*	LysoPC18:1(sn-1)	0.77*	LysoPS20:4	0.76*
LysoPI20:2	0.74*	LysoPC18:3(sn-1)	0.79*	LysoPS22:6	0.71*
LysoPS14:0	0.73*	LysoPC19:0(sn-1)	0.80*	PG36:2	0.81*
LysoPS16:0	0.79*	LysoPC20:0(sn-1)	0.75*	PS37:5	0.78*
LysoPS17:1	0.69	LysoPC20:2(sn-1)	0.73*	PA10:0/18:0	0.82*
LysoPS18:3	0.74*	LysoPC20:3(sn-1)	0.80*	PA10:0/18:1	0.68
PA18:1/20:4	0.66	PA14:0/24:5	0.77*	PA16:1/18:4	0.75*
PG40:5	0.64	PA18:0/20:5	0.66	PS18:0/22:3	0.73*
PG40:6	0.76*	PS38:1	0.81*	PS20:3/22:6	0.67
PG40:8	0.69	PS40:1	0.81		
PI18:0/17:1	0.83*	LysoPC20:4(sn-1)	0.78*		
PC37:4	0.86*	LysoPC20:5(sn-1)	0.84*		
PC39:2	0.86*	LysoPC22:6(sn-1)	0.83*		
PC40:5	0.86*	LysoPS16:1	0.71*		
PC40:8	0.72*	LysoPS17:0	0.77*		
PE35:1	0.80*	LysoPS20:5	0.72*		
PE37:3	0.77*	PC16:0/26:0	0.82*		
PE39:6	0.82*	PC39:0	0.85*		
PE42:7	0.83*	PC40:7	0.85*		
PS32:0	0.77*	PS33:0	0.73*		
PS32:1	0.79*	PS36:6	0.72*		
PS33:2	0.69	PS38:7	0.78*		
PS34:2	0.86*				
PS35:2	0.72*				

## Discussion

With the rapid development of biotechnology and understanding on lipids, the number of scientific publications on lipidomics has been increased exponentially during the last decade. Lipidomics as a tool has been used to experimentally and clinically define the lipid profiling of lung tissue and plasma and the relationship between lipid characterization and lung function. Initially, components of rat surfactant lipidomics were dynamically investigated, of which palmitoyl–myristoyl-PC and its fragments was considered as an integral component of active surfactant and correlated with respiratory rate but not with alveolar structure [14]. Telenga et al. measured more than 1500 lipid compounds in sputum of smokers with or without COPD, and found that 13 lipids differed between smokers and COPD which correlated with lower lung function and inflammation and 20 sphingolipids between smokers and non-smokers reduced after 2-month smoking cessation [15]. Our previous studies demonstrated that plasma levels of PS and lysoPS were significantly increased, while lysoPE and PE were decreased in patients with lung cancer, and firstly reported profiles of lung cancer-specific and subtype-specific lipidomics, correlated with gene expression profiles of lipid-associated enzymes and proteins [7].

Lipidomics as lipid-targeted metabolomics describes comprehensive profiles of lipid elements in multi-samples which can distinguish healthy tissue and diseased tissue as an alternative of promising biomarkers [16]. Alterations of lipidomic profiles are highly dependent upon changes of membrane structure, energy storage, and signal transduction in human disease. For example, the previous study demonstrated that 28 lipid elements co-existed in all subtypes of lung cancer as lung cancer-specific biomarkers, while levels of PG or PI were mainly reduced in squamous cell carcinomas or adenocarcinoma, and PS or PE levels were elevated in adenocarcinoma or small cell lung cancer as lung cancer subtype-specific biomarkers [7]. Chen et al. found that levels of PE elements dominantly increased in early-stages of non-small cell lung cancer and proposed those as biomarkers [17]. Values of lipidomic profiles as disease biomarkers can be increased and specialized when the concept of clinical trans-omics is introduced and applied [18]. Clinical lipidomics is a new extension of lipidomics to study lipid profiles, pathways, and networks by characterizing and quantifying the complete spectrum of lipidomes in samples of patients, and to link the lipidomics components to clinical phenomics [19]. The present study detailed clinical phenomes and converted the descriptive information into digital information using the digital evaluation score system [2, 20]. Among selected diseases in the present study, we noticed acute clinical phenomes were highly consistent and similar, while disease-associated or specific phenomes were obvious when compared to different pathogeneses.

Critical illnesses such as severe acute physical stress is often accompanied with dysfunction of immune responses and systemic metabolism that contribute to the severity of disease. SAP is one of the common initiators that can induce or worsen critical illnesses in intensive care unit and is frequently associated with high mortality and morbidity. The present study sets out to investigate clinical lipidomics in bacteria-caused SAP and discovered PA and PG as major elements (> 75% of total lipids measured) that declined in patients with SAP, while half of the increased elements were PE and lyso-lipids. Characterizations of circulating lipidomic profiles in SAP present different from non-infection acute lung injury (e.g. APE) and from acute exacerbation on basis of chronic lung jury (e.g. AECOPD). Elevated levels of lyso-lipids may be one of the major metabolites and signal pathways in SAP. Wu et al. found that there was a comprehensive increase of phosphatidylinositol and lysophospha tidylinositol levels in recovering patients who suffered from sepsis secondary to SAP, and proposed that lyso-lipids might play a role in metabolic disruptions [21]. However, the altered values of lipidomic profiles per se sound less disease specificity, since lyso-lipids were also elevated in APE (30%) or AECOPD (16%), and PA mainly declined in both APE and AECOPD.

In order to increase the disease specificity, clinical bioinformatics has been developed to integrate patient phenomes with genomic and proteomic profiles and suggested as a powerful tool to identify and validate disease-specific biomarkers and therapeutic targets [22]. For example, Chen et al. integrated clinical informatics with proteomic profiles and selected a panel of proteins for diagnosis of AECOPD [2]. Wu et al. screened AECOPD-specific panels of gene expression profiles by RNA sequencing and microarray and then correlated it with clinical phenomes dynamically [23]. Shi et al. furthermore integrated comprehensive profiles of genomics and proteomics with clinical phenomes in AECOPD and validated mechanic roles of one identified target molecule osteopontin [3]. The present study demonstrated that some of the lipid elements only showed significantly elevated levels in one of SAP, APE, or AECOPD, whereas other lipid elements showed elevated levels to varying degrees across all groups. This might have been resulted from the limited sample of patients, metabolites with less specificity, or due to the variation of severity of disease in each patient. In the study, through simulated lQTL model and integrated lipidomic and phenomic profiles we discovered disease-specific panels of lipid elements, although some of those appeared in all SAP, APE, and AECOPD patients (e.g. PE40:2/PE18:1/22:1, PE40:1/PE22:/18:1, PG30:1), some in two groups, and some only in one group. In selected disease-specific panel, lysoPG14:0, PE38:2/PE18:1/20:1, PS32:0, or lysoPS17:1 in SAP, lysoPS18:2 in APE, or PS30:0 and PE38:7/PE16:1/22:6/18:2/20:5 in AECOPD. One clinical phenotype can be associated with a variety of altered lipid elements, e.g. erythrocyte sedimentation rate which corresponded with PS32:0, lysoPS 14:0, PC 18:1/23:1, PS 40:8, PC16:0/26:0, lysoPS 20:4, and PC 18:1/23:1; PaCO_2_ (mmHg) with lysoPE 22:6 (sn-1), lysoPG14:0, PA15:0/25:0, PA 20:0/21:5, PA 17:0/13:0, and PA19:0/23:0; of which the most are down-regulated lipid elements. The abnormality of erythrocyte sedimentation rate is present in all three diseases and can be contributed with a large number of abnormal metabolites, of which altered lipid elements can be the part of mechanism. On the other hand, one altered lipid element can be corresponded to a plurality of clinical phenotypes, indicating that the lipid elements play an important role in the development of multi-phenomes. Of those, some altered lipid elements are disease-specific or phenome-specific, although the exact value as diagnostic biomarkers need to be furthermore validated as suggested [24–32].

Our present and previous data confirm that lipidomic profiles of diseases per se can differ between healthy patients and patients whom have a disease. The DESS scores were used as quantitative phenomes, and the association between lipidomics and DESS scores were captured by our “lipid-clinic” model on different groups of patients with different diseases respectively. For one category of patients, the lipids associated with severe disease state (i.e. large DESS score) would be dissimilar to those with other categories of patients. Thus, our results supplied the disease-specific lipids associated with particular disease severity, and these disease-specific lipids would be applied as potential biomarkers or therapeutic targets for given disease. Maile et al. found that changes in some lipid concentrations over time did not differ between survivors and non-survivors of patients with ARDS, while some appeared different after using multi-comparisons [33]. On the one hand, eQTL usually models the association between DNA data (e.g. sequence variants as genotype) and mRNA data (e.g. gene expression as quantitative molecular phenotype). On the other hand, this work tends to model the association between lipid data (e.g. lipidomics as molecular phenotype) and clinical data (e.g. DESS scores as quantitative phenomes). Thus, we hold an assumption that the association concept and mathematical model of conventional eQTL study can also be applied in detect the lipid-clinic association, so that, we borrowed the calculation methods from eQTL to this work. To avoid the confusion, we change the term “lipid-QTL model” to “lipid-clinic model” in revision. The modified “eQTL” analysis associated the values of individual lipid elements as patient lipid variations with index levels of each clinical phenome as patient disease phenotype. There are still urgent needs to develop more specific and accurate analytic methods to lipidomic profiles with clinical phenomes, genomics, and proteomics. It would be more helpful if the measurements of lipidomic profiles and expression of genes and proteins can be performed in the same cell bulks, or more optimally in the same single cell. The preliminary information on the clinical trans-omics was generated from the current study, although there is still a large space for improvement in future.

## Conclusions

The morbidity and mortality of critical illnesses remain high in intensive care units due to lack of understanding biomarkers and mechanisms. The present study demonstrates that lipidomic profiles of patients with acute lung diseases are different from healthy lungs, and there are also disease-specific portions of lipidomics among SAP, APE, or AECOPD. The comprehensive profiles of clinical phenomes or lipidomics are valuable in describing the disease specificity of patient phenomes and lipid elements. The combination of clinical phenomes with lipidomic profiles provides more detailed disease-specific information on panels of lipid elements when compared to the use of each separately. With integrating biological functions with disease specificity, we believe that clinical lipidomics will create a new alternative way to understand lipid-associated mechanisms of critical illnesses and develop a new category of disease-specific biomarkers and therapeutic targets.

## Additional files

**Additional file 1.** Additional tables.

**Additional file 2: Figure S1.** The map of lipidomic profiles of healthy control or patients with severe acute pneumonia, acute pulmonary embolism, or acute exacerbation of chronic pulmonary diseases. The average levels of 502 lipid elements were used and scattered from blue to red colors, indicating levels of lipid elements changes from low to high. **Figure S2.** Top 6 of the highest values of lipid elements were selected from healthy patients or patients with control, with severe acute pneumonia (SAP), acute pulmonary embolism (APE), or acute exacerbation of chronic pulmonary diseases (AECOPD). Those top 6 elements selected are independent upon statistical significance, different from Figure S4. **Figure S3.** The ROC curve of up-regulated lipid elements in severe acute pneumonia with statistical significance. **Figure S4.** The ROC curve of up-regulated lipid elements in acute pulmonary embolism with statistical significance. **Figure S5.** The ROC curve of up-regulated lipid elements in acute exacerbation of chronic pulmonary diseases with statistical significance. **Figure S6.** The ROC curve of down-regulated lipid elements in severe acute pneumonia, acute pulmonary embolism, or acute exacerbation of chronic pulmonary diseases with statistical significance.

## Data Availability

We promise that the data and materials are true, available, and reliable.

## Abbreviations

PC: phosphatidylcholine
LysoPC: lysophosphatidylcholine
Cer: ceramide
SM: sphingomyelin
PE: phosphatidylethanolamine
LysoPE: lysophosphatidylethanolamine
PA: phosphatidic acid
LysoPA: lysophosphatidic acid
PG: phosphatidyl glycerol
PS: phosphatidylserine
PI: phosphatidylinositol
LysoPS: lysophosphatide
