# Supplementary material for: Values of integration between lipidomics and clinical phenomes in patients with acute lung infection, pulmonary embolism, or acute exacerbation of chronic pulmonary diseases: a preliminary study
Source: J Transl Med. 2019 May 20;17:162. doi: 10.1186/s12967-019-1898-z (PMC6528323; doi:10.1186/s12967-019-1898-z)
Supplement: Supplementary file 1 — Additional file 1. Additional tables. [file 12967_2019_1898_MOESM1_ESM.doc]

| Supplemental Table 1: Statistical Details of integrated clinical phenomes with lipid elements in patients with severe acute pneumonia | | | |
| --- | --- | --- | --- |
| **ID** | **score** | **lipid** | **P value** |
| 2492 | nutriture | lysoPS17:1 | 0.03313735 |
| 892 | night Sweats | lysoPE 22:6 (sn-1) | 0.001394868 |
| 893 | hoarseness | lysoPE 22:6 (sn-1) | 0.001394868 |
| 894 | bronchiectasia | lysoPE 22:6 (sn-1) | 0.001394868 |
| 895 | Smoking (pack * years) | lysoPE 22:6 (sn-1) | 0.001394868 |
| 896 | P2 hyperfunction | lysoPE 22:6 (sn-1) | 0.001394868 |
| 897 | pulmonary arterial hypertension | lysoPE 22:6 (sn-1) | 0.001394868 |
| 898 | lung examination/barrel chest | lysoPE 22:6 (sn-1) | 0.001394868 |
| 899 | percussion of the lung | lysoPE 22:6 (sn-1) | 0.001394868 |
| 900 | Na(mmol/L) | lysoPE 22:6 (sn-1) | 0.001394868 |
| 901 | PaCO2(mmHg) | lysoPE 22:6 (sn-1) | 0.001394868 |
| 1671 | SaO2(%) | lysoPE 22:6 (sn-1) | 0.011552637 |
| 1916 | blood sugar（mmol/L) | lysoPE 22:6 (sn-1) | 0.017238022 |
| 2541 | coronary heart disease | lysoPE 22:6 (sn-1) | 0.03460822 |
| 787 | night Sweats | lysoPG14:0 | 0.000861727 |
| 788 | hoarseness | lysoPG14:0 | 0.000861727 |
| 789 | bronchiectasia | lysoPG14:0 | 0.000861727 |
| 790 | Smoking (pack * years) | lysoPG14:0 | 0.000861727 |
| 791 | P2 hyperfunction | lysoPG14:0 | 0.000861727 |
| 792 | pulmonary arterial hypertension | lysoPG14:0 | 0.000861727 |
| 793 | lung examination/barrel chest | lysoPG14:0 | 0.000861727 |
| 794 | percussion of the lung | lysoPG14:0 | 0.000861727 |
| 795 | Na(mmol/L) | lysoPG14:0 | 0.000861727 |
| 796 | PaCO2(mmHg) | lysoPG14:0 | 0.000861727 |
| 1021 | blood pressure | lysoPG14:0 | 0.002508924 |
| 1389 | coronary heart disease | lysoPG14:0 | 0.006315726 |
| 2144 | SaO2(%) | lysoPG14:0 | 0.02318374 |
| 2149 | blood sugar（mmol/L) | lysoPG14:0 | 0.02335835 |
| 2484 | ESR | PS32:0 | 0.03291804 |
| 2085 | nutriture | PS32:1 | 0.02173271 |
| 2736 | respiratory rate (times /min) | PS32:1 | 0.03968157 |
| 1107 | empyema | lysoPI 20:2 (sn-1) | 0.003459056 |
| 1108 | lymphadenectasis | lysoPI 20:2 (sn-1) | 0.003459056 |
| 1452 | pleural effusion | lysoPS14:0 | 0.007541542 |
| 1505 | D- dimer | lysoPS14:0 | 0.008803174 |
| 1512 | insomnia | lysoPS14:0 | 0.008916226 |
| 1573 | tired | lysoPS14:0 | 0.009643074 |
| 1584 | dysphoria | lysoPS14:0 | 0.009866435 |
| 1585 | respiratory failure | lysoPS14:0 | 0.009866435 |
| 1586 | mind | lysoPS14:0 | 0.009866435 |
| 1587 | dysphagia | lysoPS14:0 | 0.009866435 |
| 1588 | difficulty urinating or defecating | lysoPS14:0 | 0.009866435 |
| 1589 | heart visual examination | lysoPS14:0 | 0.009866435 |
| 1590 | creatinine | lysoPS14:0 | 0.009866435 |
| 1591 | HDL | lysoPS14:0 | 0.009866435 |
| 1592 | NSE | lysoPS14:0 | 0.009866435 |
| 1706 | inappetence | lysoPS14:0 | 0.012199215 |
| 1734 | lung consolidation | lysoPS14:0 | 0.012698204 |
| 1824 | Ca(mmol/L) | lysoPS14:0 | 0.0145622 |
| 1978 | ESR | lysoPS14:0 | 0.018969771 |
| 2193 | appetite | lysoPS14:0 | 0.02467988 |
| 2194 | urea | lysoPS14:0 | 0.02467988 |
| 2542 | Other chronic diseases | lysoPS14:0 | 0.03465371 |
| 2543 | increased bronchovascular shadows | lysoPS14:0 | 0.03465371 |
| 2805 | dyspnea | lysoPS14:0 | 0.04179411 |
| 2841 | K(mmol/L) | lysoPS14:0 | 0.04259847 |
| 2604 | hemameba | lysoPI19:0 (sn-1) | 0.03648257 |
| 2624 | lung consolidation | lysoPI19:0 (sn-1) | 0.0368259 |
| 2840 | lung auscultation | PA 18:1/20:4 | 0.04257414 |
| 518 | diarrhea | PG40:5 | 0.000161899 |
| 519 | lose weight | PG40:5 | 0.000161899 |
| 520 | heart rate (times /min) | PG40:5 | 0.000161899 |
| 521 | increased intracranial pressure | PG40:5 | 0.000161899 |
| 1070 | temperature | PG40:5 | 0.003068012 |
| 2559 | fever | PG40:5 | 0.03513162 |
| 784 | blood platelet | PG40:6 | 0.000855897 |
| 1124 | temperature | PG40:6 | 0.00358028 |
| 2632 | diarrhea | PG40:6 | 0.03710602 |
| 2633 | lose weight | PG40:6 | 0.03710602 |
| 2634 | heart rate (times /min) | PG40:6 | 0.03710602 |
| 2635 | increased intracranial pressure | PG40:6 | 0.03710602 |
| 2680 | expectoration | PG40:6 | 0.03796559 |
| 2750 | limitation of motion | PG40:6 | 0.03995836 |
| 2842 | hemoglobin(g/L) | PG40:6 | 0.04261336 |
| 607 | temperature | PG40:8 | 0.000323048 |
| 1179 | blood platelet | PG40:8 | 0.004202756 |
| 1514 | fever | PG40:8 | 0.008989535 |
| 1633 | diarrhea | PG40:8 | 0.010813748 |
| 1634 | lose weight | PG40:8 | 0.010813748 |
| 1635 | heart rate (times /min) | PG40:8 | 0.010813748 |
| 1636 | increased intracranial pressure | PG40:8 | 0.010813748 |
| 2435 | limitation of motion | PG40:8 | 0.03106757 |
| 2895 | Other chronic diseases | PG40:8 | 0.04445455 |
| 2896 | increased bronchovascular shadows | PG40:8 | 0.04445455 |
| 3015 | LDH | PG40:8 | 0.04812981 |
| 2023 | pleural effusion | PI 18:0/17:1 | 0.01995673 |
| 2741 | empyema | PI 18:0/17:1 | 0.03977104 |
| 2742 | lymphadenectasis | PI 18:0/17:1 | 0.03977104 |
| 2569 | CRP | PI 38:1 | 0.03550183 |
| 2992 | hemoglobin(g/L) | PI 38:1 | 0.0475259 |
| 922 | Ca(mmol/L) | PC 18:1/23:1 | 0.001609299 |
| 2553 | ESR | PC 18:1/23:1 | 0.03495242 |
| 136 | dysphoria | lysoPE19:0 | 3.94E-06 |
| 137 | respiratory failure | lysoPE19:0 | 3.94E-06 |
| 138 | mind | lysoPE19:0 | 3.94E-06 |
| 139 | dysphagia | lysoPE19:0 | 3.94E-06 |
| 140 | difficulty urinating or defecating | lysoPE19:0 | 3.94E-06 |
| 141 | heart visual examination | lysoPE19:0 | 3.94E-06 |
| 142 | creatinine | lysoPE19:0 | 3.94E-06 |
| 143 | HDL | lysoPE19:0 | 3.94E-06 |
| 144 | NSE | lysoPE19:0 | 3.94E-06 |
| 314 | inappetence | lysoPE19:0 | 3.57E-05 |
| 465 | dyspnea | lysoPE19:0 | 0.000118818 |
| 525 | lung consolidation | lysoPE19:0 | 0.000167529 |
| 812 | appetite | lysoPE19:0 | 0.000891499 |
| 813 | urea | lysoPE19:0 | 0.000891499 |
| 941 | PS scores | lysoPE19:0 | 0.001738974 |
| 968 | limitation of motion | lysoPE19:0 | 0.002066842 |
| 1018 | PaO2(mmHg) | lysoPE19:0 | 0.002477835 |
| 1061 | insomnia | lysoPE19:0 | 0.002959142 |
| 1177 | tired | lysoPE19:0 | 0.004123195 |
| 1580 | edema of lower extremity | lysoPE19:0 | 0.009803906 |
| 1581 | the thoracic compliance decreased | lysoPE19:0 | 0.009803906 |
| 1582 | PH | lysoPE19:0 | 0.009803906 |
| 2971 | Total point | lysoPE19:0 | 0.04701361 |
| 216 | empyema | lysoPI22:6 (sn-1) | 1.13E-05 |
| 217 | lymphadenectasis | lysoPI22:6 (sn-1) | 1.13E-05 |
| 538 | empyema | PA 15:0/20:5 | 0.000182534 |
| 539 | lymphadenectasis | PA 15:0/20:5 | 0.000182534 |
| 2306 | CRP | PA 15:0/20:5 | 0.02771158 |
| 2751 | Respiratory sounds in both lungs are symmetrical | PA 15:0/20:5 | 0.0400306 |
| 3010 | Past history of COPD | PA 15:0/20:5 | 0.04800401 |
| 3011 | emphysema | PA 15:0/20:5 | 0.04800401 |
| 2621 | fever | PA 14:1/20:5 | 0.03663548 |
| 2866 | empyema | PA 14:1/20:5 | 0.04330202 |
| 2867 | lymphadenectasis | PA 14:1/20:5 | 0.04330202 |
| 994 | SaO2(%) | PA 15:0/25:0 | 0.002239304 |
| 1849 | blood sugar（mmol/L) | PA 15:0/25:0 | 0.01516984 |
| 2908 | pleural effusion | PA 15:0/25:0 | 0.04499162 |
| 2920 | night Sweats | PA 15:0/25:0 | 0.04520504 |
| 2921 | hoarseness | PA 15:0/25:0 | 0.04520504 |
| 2922 | bronchiectasia | PA 15:0/25:0 | 0.04520504 |
| 2923 | Smoking (pack * years) | PA 15:0/25:0 | 0.04520504 |
| 2924 | P2 hyperfunction | PA 15:0/25:0 | 0.04520504 |
| 2925 | pulmonary arterial hypertension | PA 15:0/25:0 | 0.04520504 |
| 2926 | lung examination/barrel chest | PA 15:0/25:0 | 0.04520504 |
| 2927 | percussion of the lung | PA 15:0/25:0 | 0.04520504 |
| 2928 | Na(mmol/L) | PA 15:0/25:0 | 0.04520504 |
| 2929 | PaCO2(mmHg) | PA 15:0/25:0 | 0.04520504 |
| 937 | SaO2(%) | PA 20:0/21:5 | 0.001687227 |
| 1574 | blood sugar（mmol/L) | PA 20:0/21:5 | 0.009646548 |
| 2606 | night Sweats | PA 20:0/21:5 | 0.03650801 |
| 2607 | hoarseness | PA 20:0/21:5 | 0.03650801 |
| 2608 | bronchiectasia | PA 20:0/21:5 | 0.03650801 |
| 2609 | Smoking (pack * years) | PA 20:0/21:5 | 0.03650801 |
| 2610 | P2 hyperfunction | PA 20:0/21:5 | 0.03650801 |
| 2611 | pulmonary arterial hypertension | PA 20:0/21:5 | 0.03650801 |
| 2612 | lung examination/barrel chest | PA 20:0/21:5 | 0.03650801 |
| 2613 | percussion of the lung | PA 20:0/21:5 | 0.03650801 |
| 2614 | Na(mmol/L) | PA 20:0/21:5 | 0.03650801 |
| 2615 | PaCO2(mmHg) | PA 20:0/21:5 | 0.03650801 |
| 2501 | night Sweats | PA 17:0/13:0 | 0.0335623 |
| 2502 | hoarseness | PA 17:0/13:0 | 0.0335623 |
| 2503 | bronchiectasia | PA 17:0/13:0 | 0.0335623 |
| 2504 | Smoking (pack * years) | PA 17:0/13:0 | 0.0335623 |
| 2505 | P2 hyperfunction | PA 17:0/13:0 | 0.0335623 |
| 2506 | pulmonary arterial hypertension | PA 17:0/13:0 | 0.0335623 |
| 2507 | lung examination/barrel chest | PA 17:0/13:0 | 0.0335623 |
| 2508 | percussion of the lung | PA 17:0/13:0 | 0.0335623 |
| 2509 | Na(mmol/L) | PA 17:0/13:0 | 0.0335623 |
| 2510 | PaCO2(mmHg) | PA 17:0/13:0 | 0.0335623 |
| 2511 | pleural effusion | PA 17:0/13:0 | 0.03364782 |
| 1504 | pleural effusion | PA 19:0/23:0 | 0.008744886 |
| 1701 | SaO2(%) | PA 19:0/23:0 | 0.012089374 |
| 2344 | blood sugar（mmol/L) | PA 19:0/23:0 | 0.02890355 |
| 2601 | D- dimer | PA 19:0/23:0 | 0.03632005 |
| 2717 | night Sweats | PA 19:0/23:0 | 0.03931949 |
| 2718 | hoarseness | PA 19:0/23:0 | 0.03931949 |
| 2719 | bronchiectasia | PA 19:0/23:0 | 0.03931949 |
| 2720 | Smoking (pack * years) | PA 19:0/23:0 | 0.03931949 |
| 2721 | P2 hyperfunction | PA 19:0/23:0 | 0.03931949 |
| 2722 | pulmonary arterial hypertension | PA 19:0/23:0 | 0.03931949 |
| 2723 | lung examination/barrel chest | PA 19:0/23:0 | 0.03931949 |
| 2724 | percussion of the lung | PA 19:0/23:0 | 0.03931949 |
| 2725 | Na(mmol/L) | PA 19:0/23:0 | 0.03931949 |
| 2726 | PaCO2(mmHg) | PA 19:0/23:0 | 0.03931949 |
| 2768 | PaO2(mmHg) | PA 14:1/21:4 | 0.04041249 |
| 1221 | lung auscultation | PS40:1 | 0.004747996 |
| 2184 | chest auscultation | PS40:1 | 0.02418134 |
| 373 | tired | PS40:8 | 5.86E-05 |
| 413 | Other chronic diseases | PS40:8 | 8.85E-05 |
| 414 | increased bronchovascular shadows | PS40:8 | 8.85E-05 |
| 608 | insomnia | PS40:8 | 0.000324536 |
| 1247 | lung auscultation | PS40:8 | 0.005032687 |
| 1886 | PS scores | PS40:8 | 0.016405168 |
| 1898 | inappetence | PS40:8 | 0.01667148 |
| 1979 | appetite | PS40:8 | 0.018976276 |
| 1980 | urea | PS40:8 | 0.018976276 |
| 2204 | dysphoria | PS40:8 | 0.02473389 |
| 2205 | respiratory failure | PS40:8 | 0.02473389 |
| 2206 | mind | PS40:8 | 0.02473389 |
| 2207 | dysphagia | PS40:8 | 0.02473389 |
| 2208 | difficulty urinating or defecating | PS40:8 | 0.02473389 |
| 2209 | heart visual examination | PS40:8 | 0.02473389 |
| 2210 | creatinine | PS40:8 | 0.02473389 |
| 2211 | HDL | PS40:8 | 0.02473389 |
| 2212 | NSE | PS40:8 | 0.02473389 |
| 2299 | ESR | PS40:8 | 0.0274776 |
| 2453 | Total point | PS40:8 | 0.03186278 |
| 2698 | limitation of motion | PS40:8 | 0.03865977 |
| 2833 | chest auscultation | PS40:8 | 0.04246838 |

| Supplemental Table 2: Statistical Details of integrated clinical phenomes with lipid elements in patients with acute pulmonary embolism | | | |
| --- | --- | --- | --- |
| **ID** | **Score** | **Lipid** | **P value** |
| 715 | Other chronic diseases | PC 16:0/26:0 | 0.007501103 |
| 716 | pulmonary nodule | PC 16:0/26:0 | 0.007501103 |
| 717 | LDH | PC 16:0/26:0 | 0.007501103 |
| 718 | ESR | PC 16:0/26:0 | 0.007501103 |
| 1063 | pleural effusion | PC 16:0/26:0 | 0.019183828 |
| 1589 | Total point | PC 16:0/26:0 | 0.048458478 |
| 1376 | D- dimer | lysoPS17:0 | 0.038082571 |
| 1377 | fever | lysoPS17:0 | 0.038082571 |
| 1378 | temperature | lysoPS17:0 | 0.038082571 |
| 1396 | limitation of motion | lysoPS17:0 | 0.038865112 |
| 1397 | PS scores | lysoPS17:0 | 0.038865112 |
| 1593 | expectoration | lysoPS17:0 | 0.048615812 |
| 1318 | pleural thickening | lysoPC 22:6 (sn-1) | 0.035144494 |
| 1319 | increased intracranial pressure | lysoPC 22:6 (sn-1) | 0.035144494 |
| 1320 | urea | lysoPC 22:6 (sn-1) | 0.035144494 |
| 1168 | pleural thickening | lysoPC 19:0 (sn-1) | 0.024842269 |
| 1169 | increased intracranial pressure | lysoPC 19:0 (sn-1) | 0.024842269 |
| 1170 | urea | lysoPC 19:0 (sn-1) | 0.024842269 |
| 563 | pleural thickening | lysoPC 20:3 (sn-1) | 0.004253056 |
| 564 | increased intracranial pressure | lysoPC 20:3 (sn-1) | 0.004253056 |
| 565 | urea | lysoPC 20:3 (sn-1) | 0.004253056 |
| 808 | tiredness | lysoPC 20:5 (sn-1) | 0.010505139 |
| 405 | pleural thickening | PC 39:0 (18:0/21:0) | 0.001480812 |
| 406 | increased intracranial pressure | PC 39:0 (18:0/21:0) | 0.001480812 |
| 407 | urea | PC 39:0 (18:0/21:0) | 0.001480812 |
| 1084 | pleural thickening | lysoPC 18:0 (sn-1) | 0.02018245 |
| 1085 | increased intracranial pressure | lysoPC 18:0 (sn-1) | 0.02018245 |
| 1086 | urea | lysoPC 18:0 (sn-1) | 0.02018245 |
| 993 | edema of lower extremity | PA 14:0/24:5 | 0.015747766 |
| 994 | chest pain | PA 14:0/24:5 | 0.015747766 |
| 995 | coronary heart disease | PA 14:0/24:5 | 0.015747766 |
| 996 | nutriture | PA 14:0/24:5 | 0.015747766 |
| 909 | expectoration | PA 18:0/20:5 | 0.013653982 |
| 1293 | cough | PS38:1 | 0.032313507 |
| 1581 | pleural thickening | PS38:1 | 0.047629756 |
| 1582 | increased intracranial pressure | PS38:1 | 0.047629756 |
| 1583 | urea | PS38:1 | 0.047629756 |
| 162 | pleural thickening | PS40:1 | 8.07E-05 |
| 163 | increased intracranial pressure | PS40:1 | 8.07E-05 |
| 164 | urea | PS40:1 | 8.07E-05 |
| 533 | tchypne | PS40:1 | 0.003933353 |
| 1041 | A history of other lung diseases | lysoPE19:0 | 0.017825438 |
| 1042 | hemameba | lysoPE19:0 | 0.017825438 |
| 609 | pleural thickening | lysoPS18:2 | 0.005118652 |
| 610 | increased intracranial pressure | lysoPS18:2 | 0.005118652 |
| 611 | urea | lysoPS18:2 | 0.005118652 |
| 647 | tchypne | lysoPS18:2 | 0.005937245 |
| 91 | pleural thickening | lysoPC 22:0 (sn-1) | 1.28E-05 |
| 92 | increased intracranial pressure | lysoPC 22:0 (sn-1) | 1.28E-05 |
| 93 | urea | lysoPC 22:0 (sn-1) | 1.28E-05 |
| 1135 | tchypne | lysoPC 22:0 (sn-1) | 0.022480465 |
| 549 | pleural thickening | PA 15:0/20:5 | 0.004039808 |
| 550 | increased intracranial pressure | PA 15:0/20:5 | 0.004039808 |
| 551 | urea | PA 15:0/20:5 | 0.004039808 |
| 984 | tchypne | PA 15:0/20:5 | 0.015435472 |
| 1000 | pleural thickening | PA 14:1/20:5 | 0.01621274 |
| 1001 | increased intracranial pressure | PA 14:1/20:5 | 0.01621274 |
| 1002 | urea | PA 14:1/20:5 | 0.01621274 |
| 1569 | pleural thickening | PA 15:0/25:0 | 0.046812416 |
| 1570 | increased intracranial pressure | PA 15:0/25:0 | 0.046812416 |
| 1571 | urea | PA 15:0/25:0 | 0.046812416 |
| 1539 | pleural thickening | PA 20:0/21:5 | 0.045779857 |
| 1540 | increased intracranial pressure | PA 20:0/21:5 | 0.045779857 |
| 1541 | urea | PA 20:0/21:5 | 0.045779857 |
| 1335 | tchypne | PA 15:1/24:0 | 0.035921369 |

| Supplemental Table 3: Statistical Details of integrated clinical phenomes with lipid elements in patients with acute exacerbation of chronic pulmonary diseases | | | |
| --- | --- | --- | --- |
| **ID** | **Score** | **Lipid** | **P value** |
| 957 | hemameba | lysoPI 22:4 (sn-1) | 0.025015402 |
| 1097 | respiratory rate (times /min) | lysoPI 22:4 (sn-1) | 0.032055509 |
| 1098 | albumin（g/L) | lysoPI 22:4 (sn-1) | 0.032055509 |
| 1100 | neutrophile granulocyte | lysoPI 22:4 (sn-1) | 0.032055509 |
| 453 | reduced exercise tolerance | lysoPS22:6 | 0.009624994 |
| 454 | P2 hyperfunction | lysoPS22:6 | 0.009624994 |
| 455 | lung examination/barrel chest | lysoPS22:6 | 0.009624994 |
| 456 | CEA | lysoPS22:6 | 0.009624994 |
| 946 | wheezing rale | lysoPS22:6 | 0.024708254 |
| 104 | A history of other lung diseases | lysoPC 17:1 (sn-1) | 0.000245651 |
| 105 | Other chronic diseases | lysoPC 17:1 (sn-1) | 0.000245651 |
| 436 | hemameba | lysoPI 20:0 (sn-1) | 0.008720719 |
| 790 | hemameba | lysoPS20:4 | 0.017758143 |
| 1258 | ESR | lysoPS20:4 | 0.040468448 |
| 1023 | reduced exercise tolerance | lysoPG15:0 | 0.028510341 |
| 1024 | P2 hyperfunction | lysoPG15:0 | 0.028510341 |
| 1025 | lung examination/barrel chest | lysoPG15:0 | 0.028510341 |
| 1026 | CEA | lysoPG15:0 | 0.028510341 |
| 1344 | wheezing rale | lysoPG15:0 | 0.044749237 |
| 485 | hemameba | PS37:5 | 0.010870657 |
| 1172 | A history of other lung diseases | PA 10:0/18:1 | 0.035148862 |
| 1173 | Other chronic diseases | PA 10:0/18:1 | 0.035148862 |
| 1130 | K(mmol/L) | PS 20:3/22:6 | 0.033176152 |
| 930 | albumin（g/L) | PC 18:1/23:1 | 0.024047618 |
| 931 | respiratory rate (times /min) | PC 18:1/23:1 | 0.024047618 |
| 933 | neutrophile granulocyte | PC 18:1/23:1 | 0.024047618 |
| 1192 | ESR | PC 18:1/23:1 | 0.036406648 |
| 216 | γ一GT | lysoPE19:0 | 0.001443128 |
| 1395 | pulmonary nodule | PA 15:0/20:5 | 0.047462319 |
| 943 | pulmonary nodule | PA 14:1/20:5 | 0.024606791 |
| 1297 | tiredness | PA 15:0/25:0 | 0.042230335 |
| 1298 | dyspnea | PA 15:0/25:0 | 0.042230335 |
| 1299 | pulmonary embolism | PA 15:0/25:0 | 0.042230335 |
| 1106 | wheeze | PA 20:0/21:5 | 0.03240129 |
| 1243 | can't lie down at night | PA 20:0/21:5 | 0.039625967 |
| 1413 | tiredness | PA 20:0/21:5 | 0.048331171 |
| 1414 | dyspnea | PA 20:0/21:5 | 0.048331171 |
| 1415 | pulmonary embolism | PA 20:0/21:5 | 0.048331171 |
| 883 | Increased bronchovascular shadows | PA 17:0/13:0 | 0.022564891 |
| 1122 | History of asthma | PA 17:0/13:0 | 0.032782511 |
| 1123 | lose weight | PA 17:0/13:0 | 0.032782511 |
| 1124 | NSE | PA 17:0/13:0 | 0.032782511 |
| 1128 | coronary heart disease | PA 17:0/13:0 | 0.033009947 |
| 1341 | blood sugar（mmol/L) | PA 17:0/13:0 | 0.044345333 |
| 312 | hemoptysis | PA 16:0/18:3 | 0.004504491 |
| 845 | edema of lower extremity | PA 16:0/18:3 | 0.020908457 |
| 917 | tiredness | PA 16:0/18:3 | 0.023678403 |
| 918 | dyspnea | PA 16:0/18:3 | 0.023678403 |
| 919 | pulmonary embolism | PA 16:0/18:3 | 0.023678403 |
| 872 | wheeze | PA 19:0/22:5 | 0.021994496 |
| 462 | hemoptysis | PA 18:4/19:1 | 0.009743245 |
| 892 | tiredness | PA 18:4/19:1 | 0.022923135 |
| 893 | dyspnea | PA 18:4/19:1 | 0.022923135 |
| 894 | pulmonary embolism | PA 18:4/19:1 | 0.022923135 |
| 1191 | edema of lower extremity | PA 18:4/19:1 | 0.036394774 |
| 1256 | blood pressure | PA 18:4/19:1 | 0.040301602 |
