# Supplementary material for: Values of integration between lipidomics and clinical phenomes in patients with acute lung infection, pulmonary embolism, or acute exacerbation of chronic pulmonary diseases: a preliminary study
Source: J Transl Med. 2019 May 20;17:162. doi: 10.1186/s12967-019-1898-z (PMC6528323; doi:10.1186/s12967-019-1898-z)
Supplement: Supplementary file 2 — Additional file 2: Figure S1. The map of lipidomic profiles of healthy control or patients with severe acute pneumonia, acute pulmonary embolism, or acute exacerbation of chronic pulmonary diseases. The average levels of 502 lipid elements were used and scattered from blue to red colors, indicating levels of lipid elements changes from low to high. Figure S2. Top 6 of the highest values of lipid elements were selected from healthy patients or patients with control, with severe acute pneumonia (SAP), acute pulmonary embolism (APE), or acute exacerbation of chronic pulmonary diseases (AECOPD). Those top 6 elements selected are independent upon statistical significance, different from Figure S4. Figure S3. The ROC curve of up-regulated lipid elements in severe acute pneumonia with statistical significance. Figure S4. The ROC curve of up-regulated lipid elements in acute pulmonary embolism with statistical significance. Figure S5. The ROC curve of up-regulated lipid elements in acute exacerbation of chronic pulmonary diseases with statistical significance. Figure S6. The ROC curve of down-regulated lipid elements in severe acute pneumonia, acute pulmonary embolism, or acute exacerbation of chronic pulmonary diseases with statistical significance. [file 12967_2019_1898_MOESM2_ESM.pot]

## Slide 1
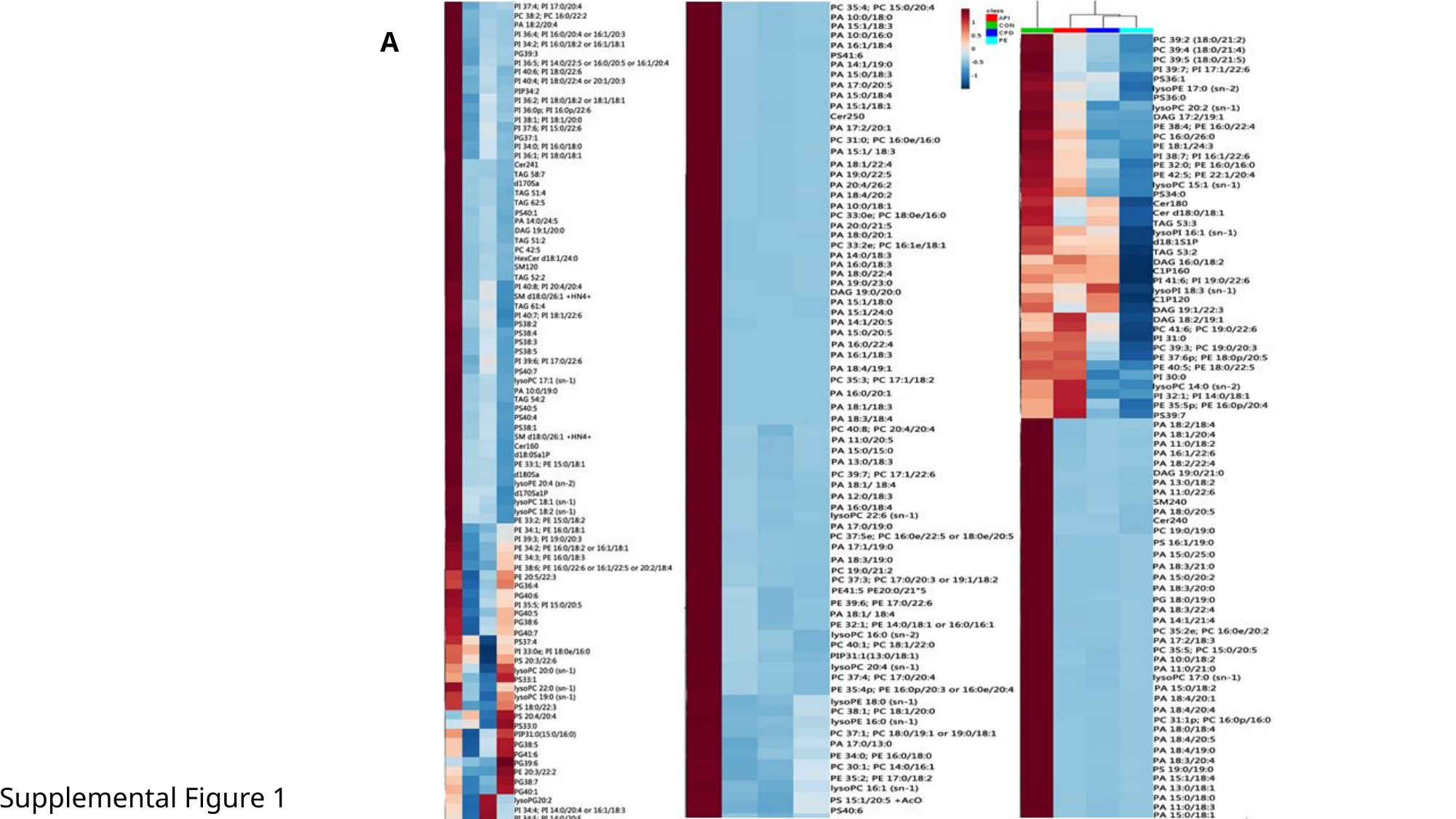

A
Supplemental Figure 1

## Slide 2
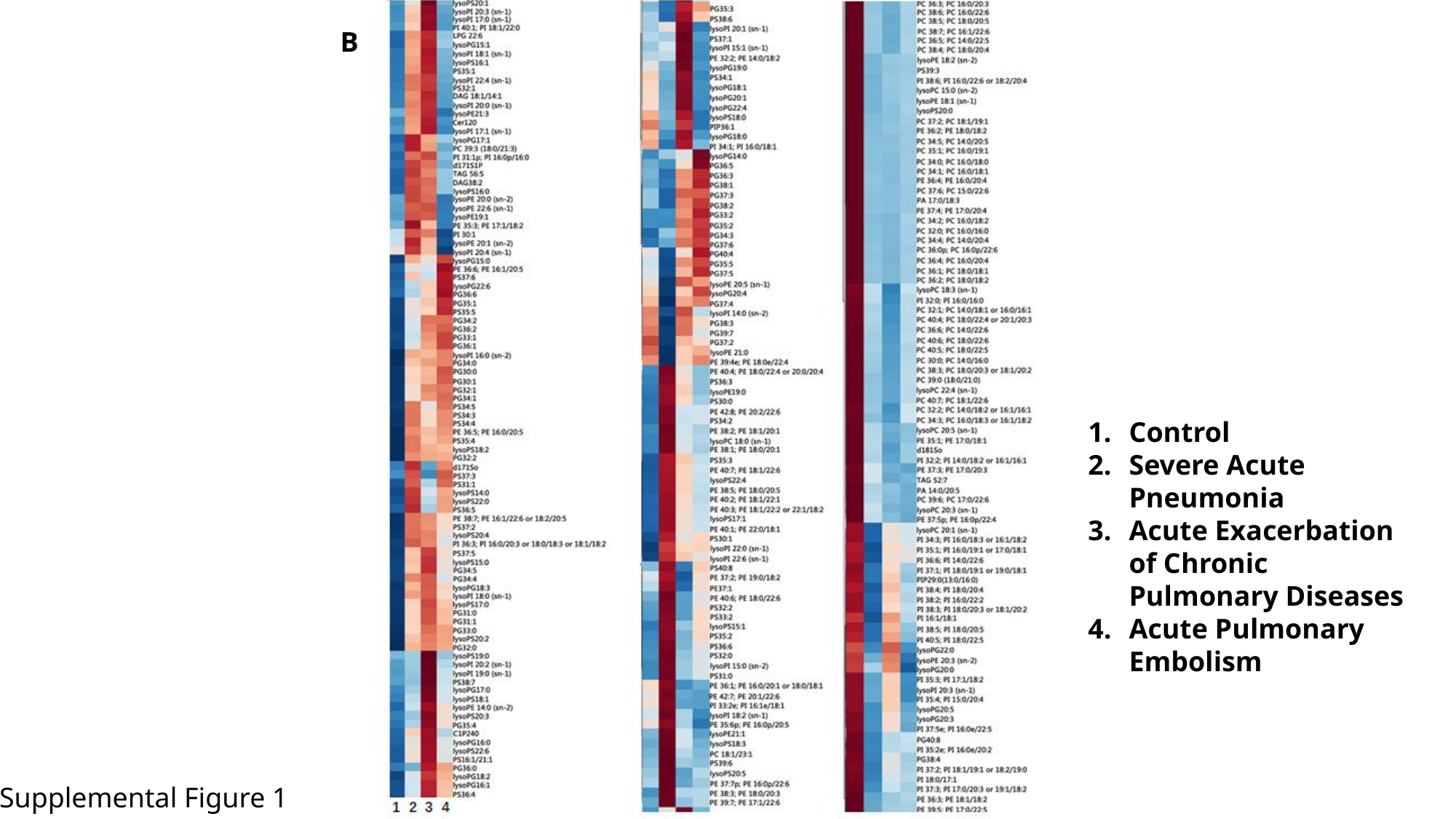

B
Control
Severe Acute Pneumonia
Acute Exacerbation of Chronic Pulmonary Diseases
Acute Pulmonary Embolism
Supplemental Figure 1

## Slide 3
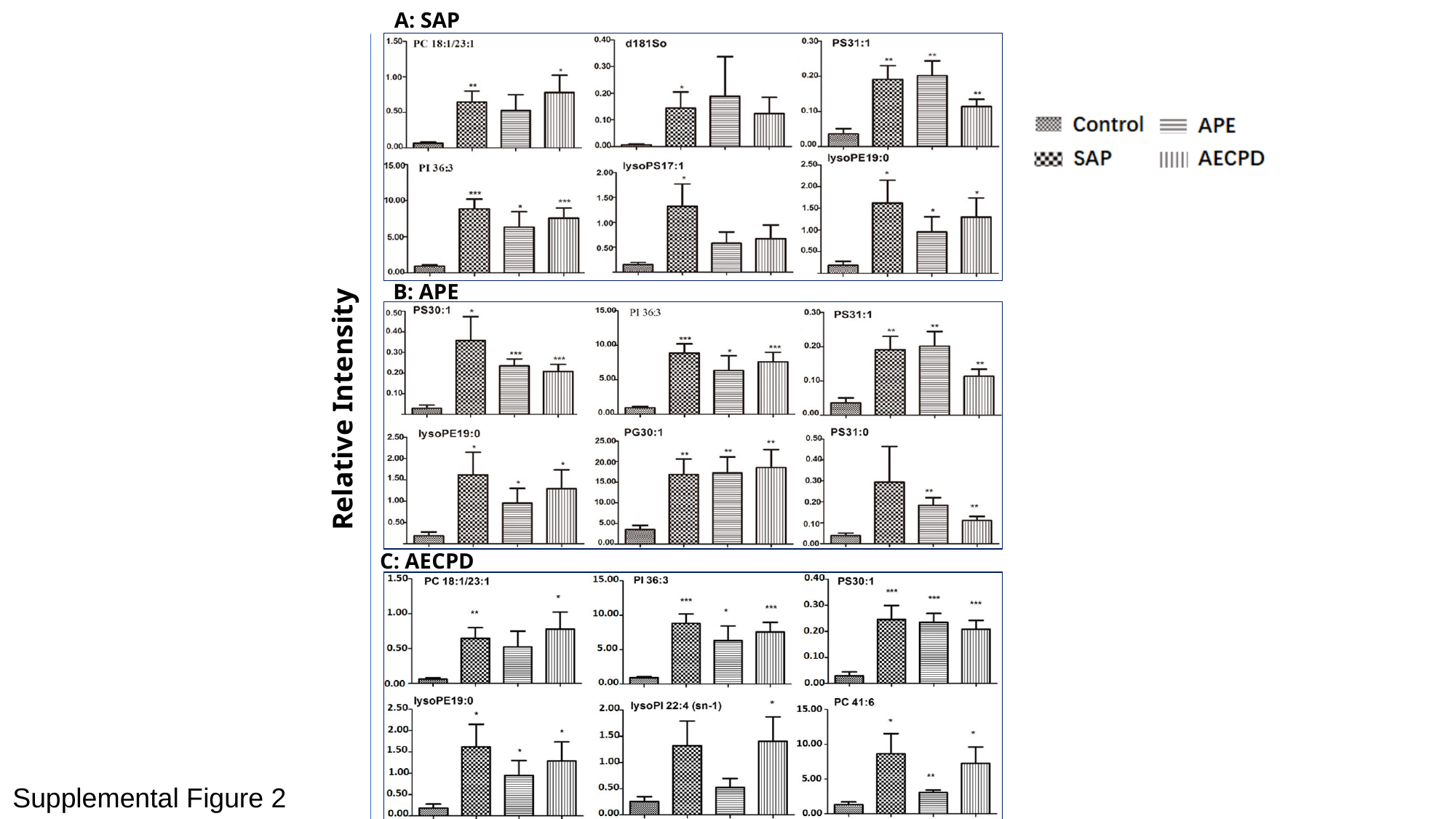

A: SAP
B: APE
Relative Intensity
C: AECPD
Supplemental Figure 2

## Slide 4
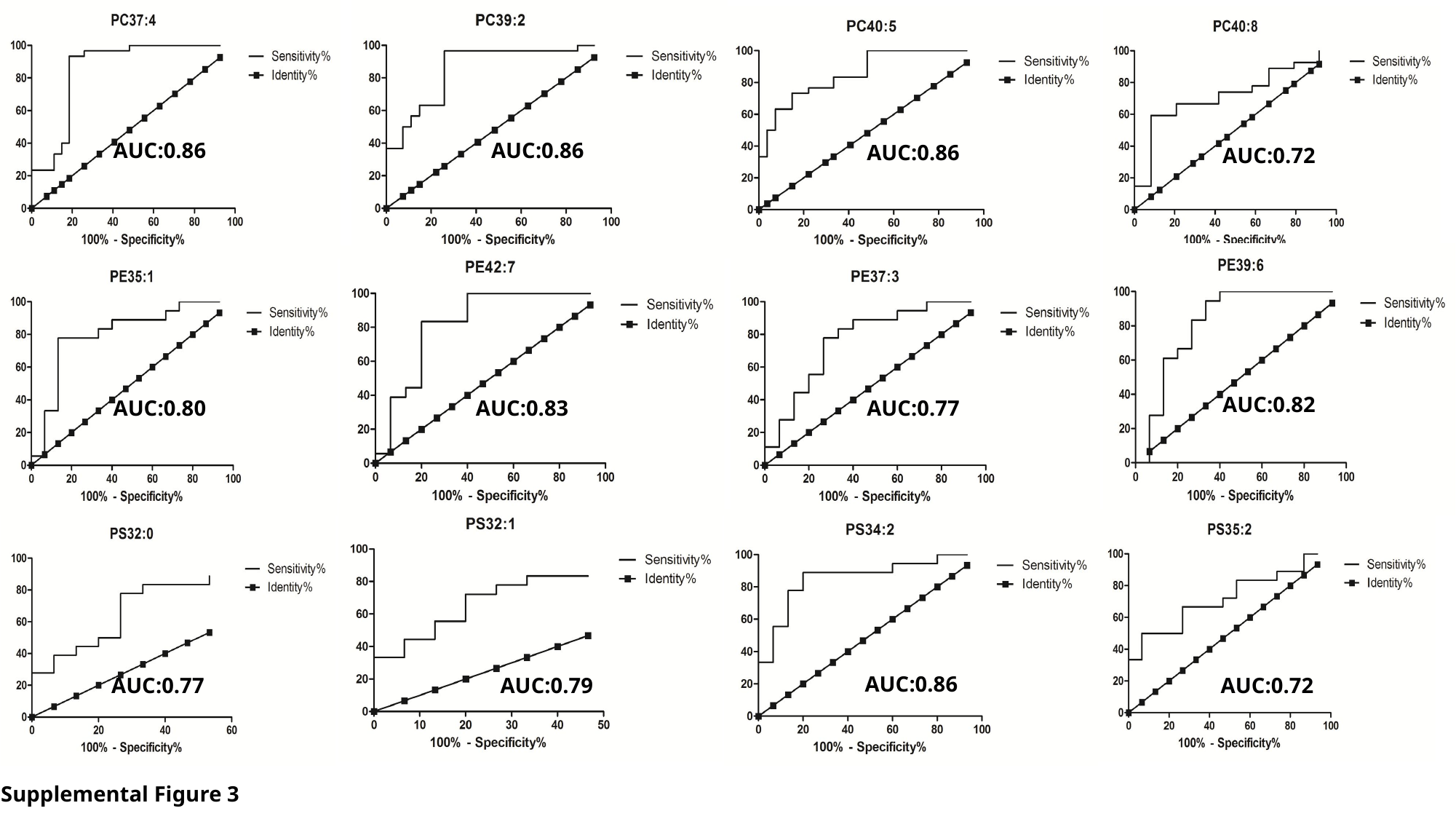

AUC:0.86
AUC:0.86
AUC:0.86
AUC:0.72
AUC:0.82
AUC:0.80
AUC:0.83
AUC:0.77
AUC:0.86
AUC:0.77
AUC:0.79
AUC:0.72
Supplemental Figure 3

## Slide 5
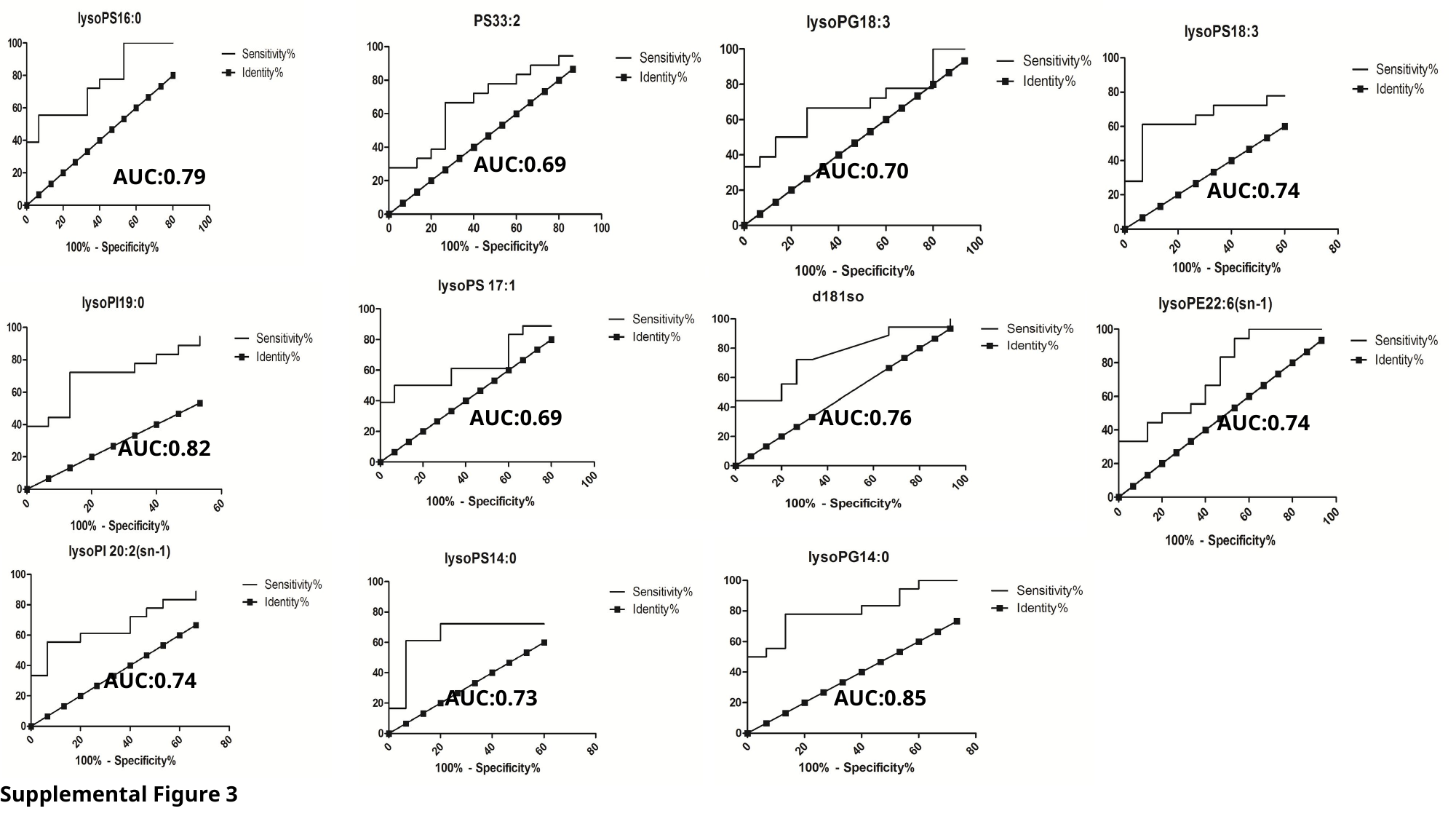

AUC:0.69
AUC:0.70
AUC:0.79
AUC:0.74
AUC:0.69
AUC:0.76
AUC:0.74
AUC:0.82
AUC:0.74
AUC:0.73
AUC:0.85
Supplemental Figure 3

## Slide 6
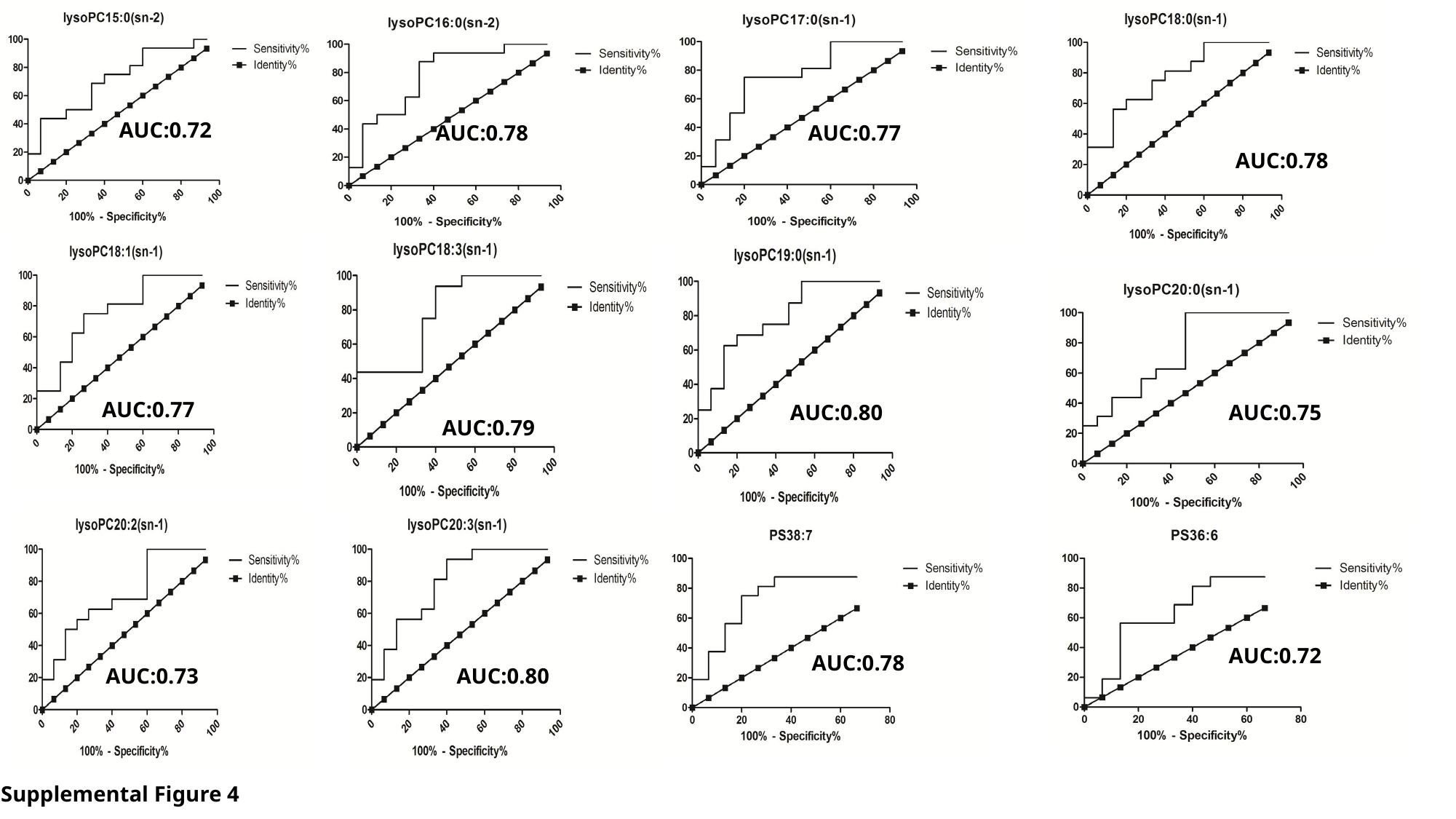

AUC:0.72
AUC:0.78
AUC:0.77
AUC:0.78
AUC:0.77
AUC:0.80
AUC:0.75
AUC:0.79
AUC:0.72
AUC:0.78
AUC:0.73
AUC:0.80
Supplemental Figure 4

## Slide 7
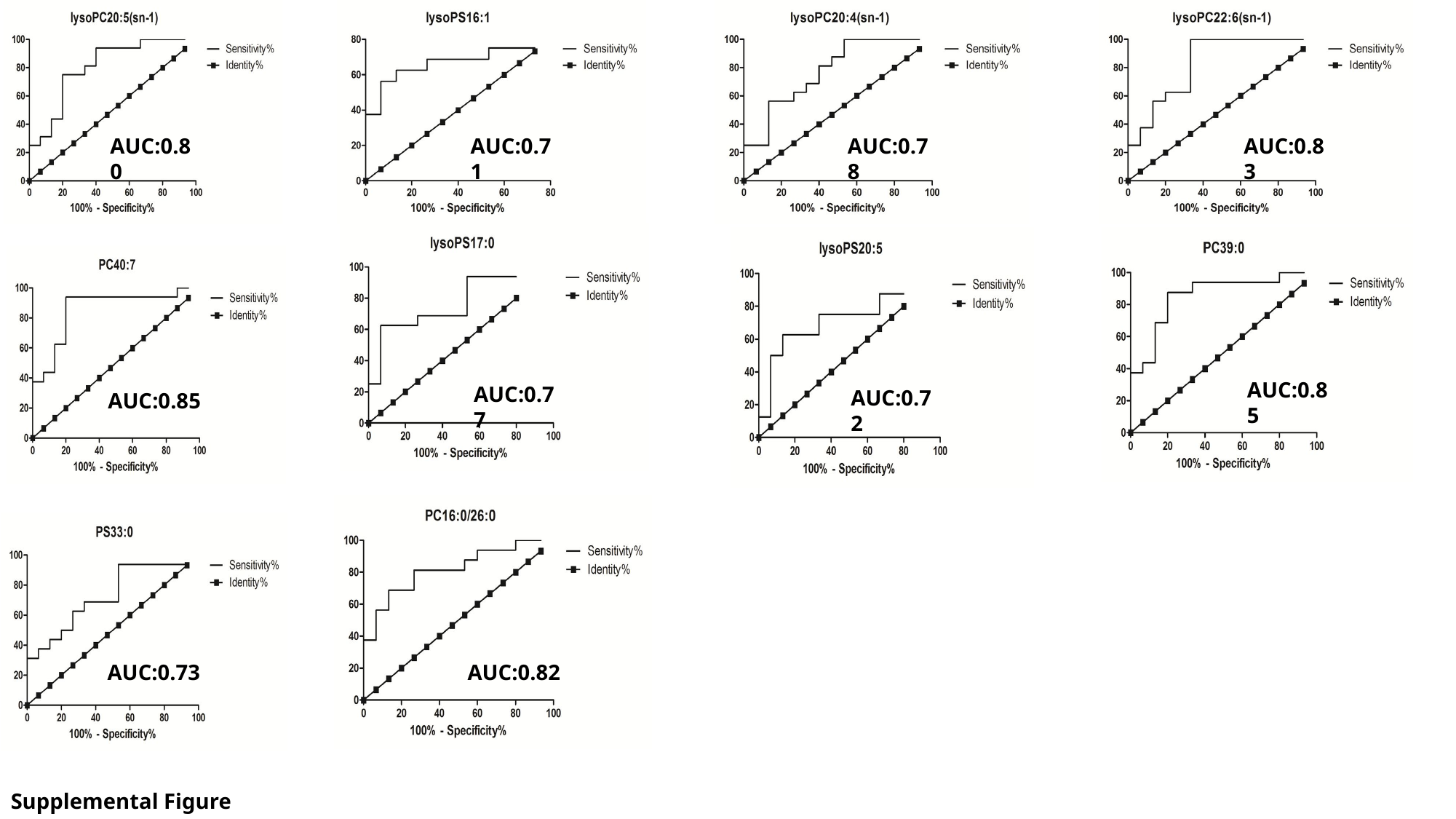

AUC:0.80
AUC:0.71
AUC:0.78
AUC:0.83
AUC:0.85
AUC:0.77
AUC:0.72
AUC:0.85
AUC:0.73
AUC:0.82
Supplemental Figure 4

## Slide 8
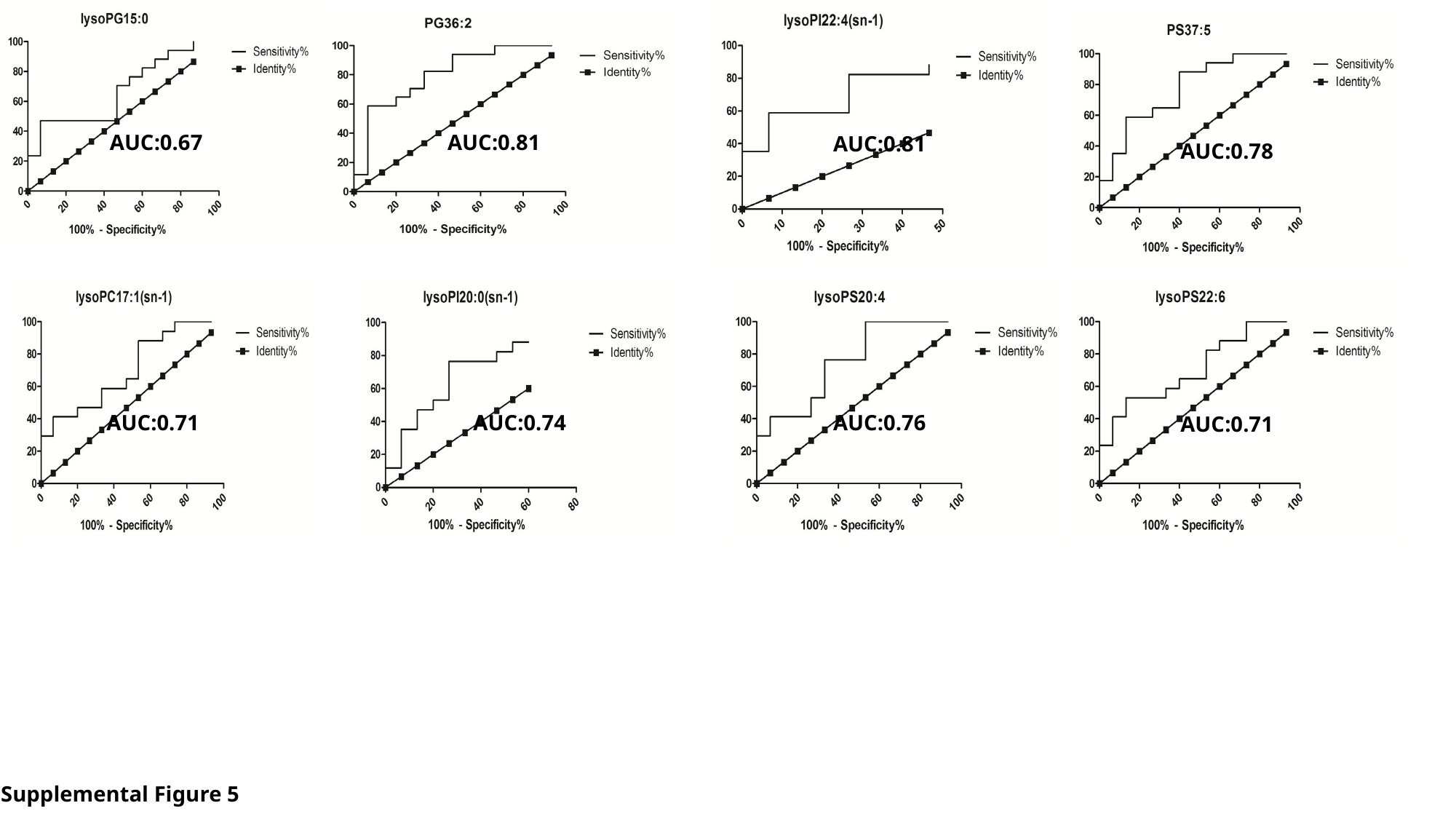

AUC:0.67
AUC:0.81
AUC:0.81
AUC:0.78
AUC:0.71
AUC:0.74
AUC:0.76
AUC:0.71
Supplemental Figure 5

## Slide 9
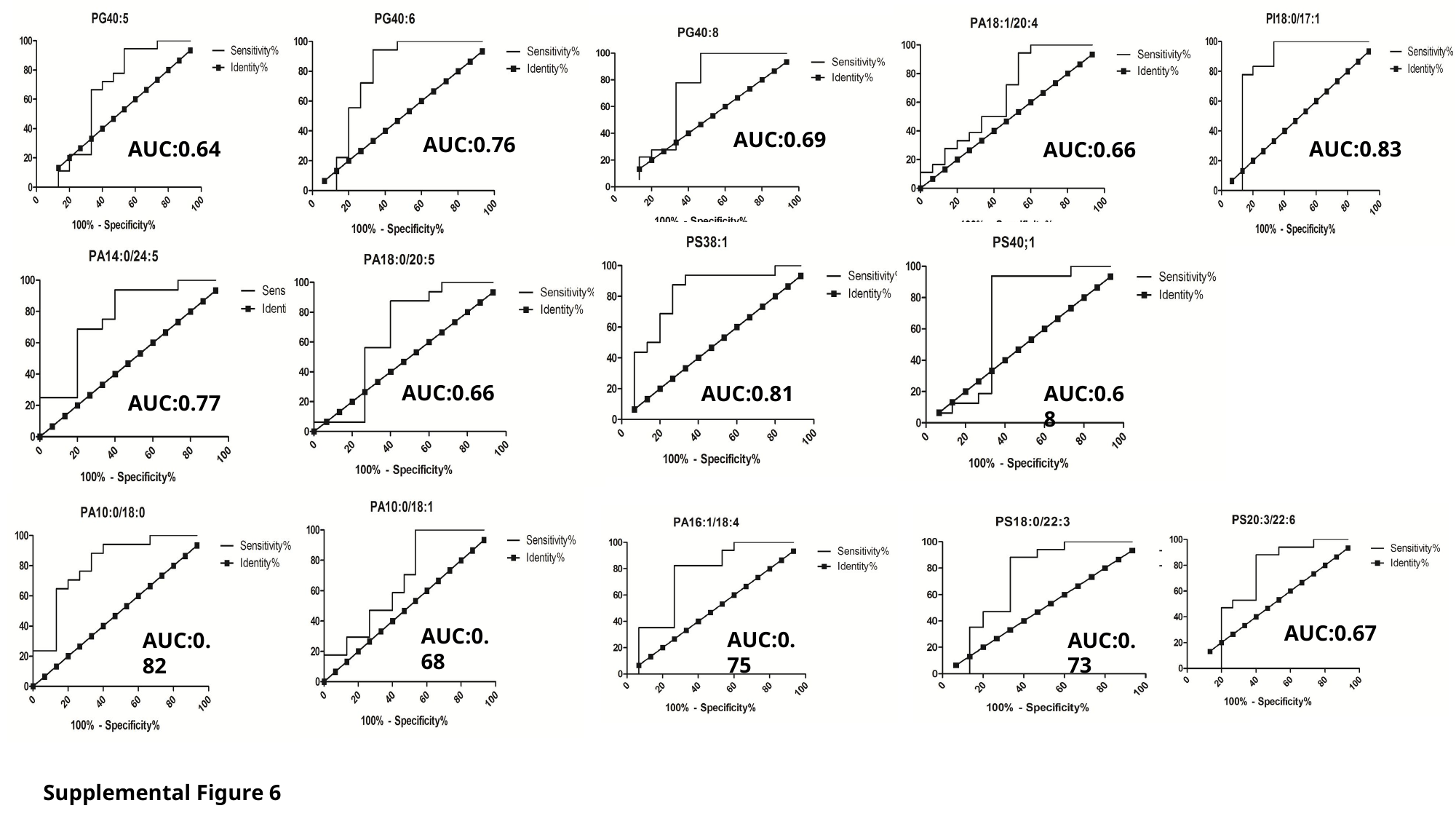

AUC:0.69
AUC:0.76
AUC:0.64
AUC:0.83
AUC:0.66
AUC:0.66
AUC:0.81
AUC:0.68
AUC:0.77
AUC:0.67
AUC:0.68
AUC:0.75
AUC:0.73
AUC:0.82
Supplemental Figure 6
